# The incidence rate of planned and emergency physical health hospital admissions in people diagnosed with severe mental illness: a cohort study

**DOI:** 10.1017/S0033291722002811

**Published:** 2023-09

**Authors:** Naomi Launders, Joseph F. Hayes, Gabriele Price, Louise Marston, David P. J. Osborn

**Affiliations:** 1 Division of Psychiatry, UCL. 6th Floor Maple House, 149 Tottenham Court Road, London W1T 7NF, UK; 2 Camden and Islington NHS Foundation Trust, St Pancras Hospital, 4 St Pancras Way, London, NW1 0PE, UK; 3 Department of Health and Social Care, Office for Health Improvement and Disparities, Wellington House, 133-155 Waterloo Road, London SE1 8UG, UK; 4 Department of Primary Care and Population Health, UCL, Rowland Hill Street, NW3 2PF, London, UK

**Keywords:** Bipolar disorder, hospital utilisation, physical health, schizophrenia, severe mental illness

## Abstract

**Background:**

People with severe mental illness (SMI) have more physical health conditions than the general population, resulting in higher rates of hospitalisations and mortality. In this study, we aimed to determine the rate of emergency and planned physical health hospitalisations in those with SMI, compared to matched comparators, and to investigate how these rates differ by SMI diagnosis.

**Methods:**

We used Clinical Practice Research DataLink Gold and Aurum databases to identify 20,668 patients in England diagnosed with SMI between January 2000 and March 2016, with linked hospital records in Hospital Episode Statistics. Patients were matched with up to four patients without SMI. Primary outcomes were emergency and planned physical health admissions. Avoidable (ambulatory care sensitive) admissions and emergency admissions for accidents, injuries and substance misuse were secondary outcomes. We performed negative binomial regression, adjusted for clinical and demographic variables, stratified by SMI diagnosis.

**Results:**

Emergency physical health (aIRR:2.33; 95% CI 2.22–2.46) and avoidable (aIRR:2.88; 95% CI 2.60–3.19) admissions were higher in patients with SMI than comparators. Emergency admission rates did not differ by SMI diagnosis. Planned physical health admissions were lower in schizophrenia (aIRR:0.80; 95% CI 0.72–0.90) and higher in bipolar disorder (aIRR:1.33; 95% CI 1.24–1.43). Accident, injury and substance misuse emergency admissions were particularly high in the year after SMI diagnosis (aIRR: 6.18; 95% CI 5.46–6.98).

**Conclusion:**

We found twice the incidence of emergency physical health admissions in patients with SMI compared to those without SMI. Avoidable admissions were particularly elevated, suggesting interventions in community settings could reduce hospitalisations. Importantly, we found underutilisation of planned inpatient care in patients with schizophrenia. Interventions are required to ensure appropriate healthcare use, and optimal diagnosis and treatment of physical health conditions in people with SMI, to reduce the mortality gap due to physical illness.

## Introduction

People with severe mental illness (SMI) have poorer physical health (Launders, Hayes, Price, & Osborn, 2022), a higher rate of non-mental health hospitalisations (Germack, Caron, Solomon, & Hanrahan, 2018; Jansen, van Schijndel, van Waarde, & van Busschbach, 2018), and a higher risk of mortality than the general population (Hayes, Marston, Walters, King, & Osborn, 2017; Walker, McGee, & Druss, 2015). A recent meta-analysis found elevated rates of non-mental health admissions in people with SMI, even when controlling for underlying physical health (Ronaldson et al., 2020), suggesting that increased prevalence of physical health conditions is not the only driver of elevated non-mental health admissions in those with SMI.

Barriers to timely preventative care and to services aimed at the management of chronic disease, may lead to increased emergency admissions. Barriers to appropriate physical health care for those with SMI include healthcare-related factors such as diagnostic overshadowing resulting in delayed diagnosis of physical health conditions, and fragmented healthcare systems hampering a patient's ability to access appropriate and timely care, as well as patient-level factors such as cognitive impairment, reduced motivation or social isolation reducing healthcare access. There is also evidence that patients with SMI are at increased risk of 30-day readmissions for physical health conditions (Germack et al., 2018; Jansen et al., 2018), suggesting treatment failure and suboptimal follow-up care and management may be responsible for some of the increased emergency hospital use in this population. Understanding the use of planned and emergency hospitalisation is therefore important to implement appropriate actions, aimed at providing accessible physical health services, reducing emergency admissions with the aim of reducing premature mortality in people living with SMI.

A subset of emergency admissions is thought to be avoidable if appropriate disease management services are in place (Purdy, Griffin, Salisbury, & Sharp, 2009). In the general population, analyses of these admissions have been used to identify subsets of patients for whom interventions in primary care may impact their emergency hospital use (Orlowski et al., 2021). In the context of people with SMI, previous studies have found elevated rates of avoidable admissions (Davydow et al., 2016; Lin, Huang, Chen, & Chen, 2011), but few if any have put this in the context of emergency admissions for other causes.

While there is evidence that non-mental health admissions are elevated in those with SMI, there is a need to investigate the contribution of physical health admissions separately from accident and injury admissions, and avoidable admissions in comparison to emergency admissions for other causes. Furthermore, there is a paucity of evidence on the impact of having SMI on planned *v.* emergency physical health admissions. The objective of this cohort study was to determine the rate of emergency and planned physical health hospitalisations in those with SMI, compared to matched comparators, and to investigate how these rates differ by SMI diagnosis.

## Methods

### Population

We identified a cohort of patients with SMI, and a comparator cohort without SMI, who were registered with a primary care practice in England from the Clinical Practice Research Datalink (CPRD) Aurum and Gold databases (Herrett et al., 2015; Wolf et al., 2019). At the time of this study, these databases held deidentified primary care medical records for over 39 million patients in the UK. We defined patients with a diagnosis of SMI using medical codes for schizophrenia, bipolar disorder, or other non-affective psychotic illnesses (online Supplementary Table S1). Patients with SMI were matched to individuals who had never had a diagnosis of SMI on sex, five-year age band, primary care practice and year of primary care practice registration to comparators. Exact matching was performed by CPRD prior to receipt of the dataset and comparators were matched at least 1:1 and up to 1:4 based on the availability of comparators. Hospitalisation data were available from 1 April 2000 to 31 March 2017, we therefore included patients with a first diagnosis of SMI between 1 April 2000 and 31 March 2016 to allow for at least one year's follow up. The index date was the date of diagnosis, or for comparators, the date that their matched patient with SMI was diagnosed. We excluded patients under the age of 18 or over 100 with less than one year of active follow up at index. Ethical approval for this study was obtained from the Independent Scientific Advisory Committee of CPRD (protocol no. 18_288).

### Data linkage

We linked data from CPRD to Hospital Episode Statistics (HES) Admitted Patient Care (APC) data for hospital activity HES APC contains details of admissions to hospitals in England where care is paid for by the National Health Service (NHS). The main purpose of HES is to inform service provision and to manage reimbursements within the NHS system (Herbert, Wijlaars, Zylbersztejn, Cromwell, & Hardelid, 2017). We also linked data from the Office for National Statistics to obtain the 2015 Index of Multiple Deprivation (IMD) (Office for National Statistics, 2015). The 2015 IMD provides a relative measure of deprivation across small areas in England as calculated in 2015. The IMD quintile was then determined for patients in CPRD based on patient postcode. We compared patients eligible for linkage to those who were not eligible to assess linkage bias.

### Study follow up

For the cohort of patients with SMI, follow up began the day following the first diagnosis of SMI received in primary care. For the comparator population, we used the same start date as their matched case. Patients were followed up from this date until their CPRD record ended or to the end of HES data; 31 March 2017.

### Outcomes

The primary outcomes were planned and emergency hospital admissions for physical health conditions. Secondary outcomes were emergency admissions for accidents, injuries, and substance misuse, and for avoidable admissions, due to ambulatory care sensitive conditions (ACSC), a list of conditions deemed potentially preventable with appropriate primary care (Purdy et al., 2009). We defined admissions as spells of continuous hospitalisation in a single hospital. We excluded hospital spells where a patient was transferred from another hospital (1.8% of admissions) or where the source of admission was not given (0.1%). The reason for admission was identified based on ICD-10 codes for the primary diagnosis of the first episode of care within a spell. We excluded maternity admissions, and admissions that were regular repeat attendances, for example for regular cancer treatment or renal dialysis, or admissions where the primary diagnosis was related to mental health, or due to congenital conditions. We classified remaining admissions as due to ‘physical health’, ‘accident, injury and substance misuse’, or ‘other cause’ (online Supplementary Table S2).

### Covariates

Covariates were derived from primary care and LSOA data. We included age, sex, ethnicity, region and IMD as potential confounders based on previous literature (Busby, Purdy, & Hollingworth, 2017b; Cournane et al., 2015; Huntley et al., 2014; Petersen, Kandt, & Longley, 2021). We defined age as age at index based on year of birth as a continuous variable. Sex and ethnicity were reported as recorded in the patient's primary care medical records. Ethnicity was grouped based on the UK 2011 Census Ethnic Group categories (Office for National Statistics, 2014): ‘Asian’, ‘Black’, ‘Mixed’, ‘White’ or ‘Other’. Where multiple ethnicities existed for an individual, we selected the most frequent, and where frequencies were equal, the most recent ever recorded. Region was based on primary care practice postcode, and IMD was based on patient postcode and, where this was missing, the primary care practice postcode.

We also investigated physical health, substance misuse, smoking and obesity as potential confounders. Underlying physical health conditions and multimorbidity are key drivers of admissions, while substance misuse, obesity and smoking may increase the severity or result in poor self-management of physical health conditions and may be precursors to physical disease. We included a count of 24 physical health conditions, defined from code lists for the Elixhauser and Charlson Comorbidity Index (Launders et al., 2022; Metcalfe et al., 2019). For investigation of avoidable admissions, angina was also included, defined using the Caliber code list (https://www.caliberresearch.org/portal/codelists). We defined alcohol and drug misuse using the code lists for the Elixhauser comorbidity index (Metcalfe et al., 2019). We categorised smoking status as never-smoker, ex-smoker, or current smoker using medical code lists, taking the most recent category prior to index, and recording any never-smokers with a historical code for smoking as ex-smokers. We took the most recent BMI prior to index, categorised as obese (BMI ⩾ 30), overweight (BMI 25 to 29.9), healthy weight (BMI 18.5 to 24.9) or underweight (BMI < 18.5), derived from the recording of obesity, BMI, and BMI calculated from weight and height recording.

### Missing data

We coded patients with missing smoking or BMI data as never-smoker and normal range BMI respectively, as general practitioners are less likely to record values that are within the normal range (Hippisley-Cox & Coupland, 2013; Marston, Nazareth, Petersen, Walters, & Osborn, 2014). We coded ethnicity as White ethnic group where this variable was missing. This approach is in line with previous research using primary care data, which suggests that more than 93% of individuals without ethnicity recorded are from a white ethnic group (Hippisley-Cox et al., 2008). Furthermore, sensitivity analyses comparing physical health outcomes using this method found similar effect estimates as using multiple imputations (Launders et al., 2022).

### Analysis

As the distribution of admissions within the study population was ‘over dispersed’, with many zero counts, we considered the use of Poisson, negative binomial and zero-inflated models in the analysis. We found little improvement between negative binomial and zero-inflated negative binomial models (online Supplementary Table S3) and therefore used negative binomial regression for all outcomes, with sandwich standard errors to account for clustering by primary care practice.

We calculated crude incidence rate ratios (IRR) of all hospital outcomes stratified by SMI diagnosis (schizophrenia, bipolar disorder, other psychoses), then calculated adjusted IRR (aIRR), first adjusting for demographic variables (age, sex, ethnicity, region, IMD quintile, calendar year); then health risk factor variables (BMI category, smoking, alcohol misuse, drug misuse) and a count of physical health conditions as recorded in primary care.

For the analysis of avoidable admissions, we additionally adjusted for diagnosis of each separate chronic condition in the ACSC definition (angina, asthma, COPD, congestive heart failure, deficiency anaemia, hypertension, diabetes, and neurological disease) as recorded in primary care and a count of the remaining physical health conditions not included in the ACSC definition.

For all outcomes, we calculated *E* values to assess the potential for residual confounding (VanderWeele & Ding, 2017). We reported this study according to the Strengthening the Reporting of Observational Studies in Epidemiology checklist (Strobe Checklist) (von Elm et al., 2007).

### Sensitivity analyses

We conducted five sensitivity analyses which were defined a priori. We investigated admissions in the first year post SMI diagnosis, due to the potential for different healthcare use due to increased physical health monitoring or less stable mental health compared to other time points. We investigated the effect of covariates updated at one-year post-diagnosis, to account for potential increased capture of physical health conditions around the time of SMI diagnosis. We limited the population to those with at least one year of registration prior to the start date, to investigate the impact of possible incomplete baseline data, and reran the main analysis with individual physical health conditions rather than a count to investigate the potential of residual confounding. Finally, we ran the models to account for the use of primary (online Supplementary Table S4), and secondary (planned and emergency non-mental health admissions) care use in the year prior to the start date. A final post-hoc sensitivity analysis was performed to assess the impact of using a zero-inflated negative binomial model for the primary outcomes.

## Results

We identified 118468 patients eligible for linkage with HES, of which 23415 did not meet our inclusion/exclusion criteria. A further 108 had multiple records in CPRD for one HES record and 2996 could not be matched at least 1:1 (online Supplementary Fig. S1). The final cohort consisted of 20568 people with SMI and 71 381 matched comparators (Table 1). Patients eligible for HES linkage were similar in respect to SMI diagnosis, age at start of follow up, sex, BMI, smoking and multimorbidity to those which were ineligible (online Supplementary Table S5). A total of 67.58% of patients could be matched 1:4, 25.79% 1:3, 5.49% 1:2 and 1.14% 1:1.

**Table 1.** Demographic and clinical variables in those with and without SMI

	SMI	No SMI
*n* (%)	20 568 (22.37)	71 381 (77.63)
Data source		
Gold (%)	9566 (46.51)	33 879 (47.46)
Aurum (%)	11 002 (53.49)	37 502 (52.54)
Diagnosis (%)		
Schizophrenia	4553 (22.14)	–
Bipolar	7455 (36.25)	–
Other	8560 (41.62)	–
Age at index^a^ [median (IQR)]	41.04 [29.36–56.59]	41.79 [29.82–57.02]
Female (%)	10 060 (48.91)	34 761 (48.70)
Ethnicity^b^ (%)		
Asian	1077 (5.24)	3925 (5.50)
Black	998 (4.85)	2143 (3.00)
Mixed	237 (1.15)	514 (0.72)
Other	507 (2.46)	1741 (2.44)
White	17 749 (86.29)	63 058 (88.34)
Region		
East Midlands	664 (3.23)	2415 (3.38)
East of England	1740 (8.46)	6255 (8.76)
London	4162 (20.24)	13 852 (19.41)
North East	407 (1.98)	1471 (2.06)
North West	2720 (13.22)	9465 (13.26)
South Central	2837 (13.79)	9812 (13.75)
South East Coast	1996 (9.70)	6861 (9.61)
South West	2893 (14.07)	9991 (14.00)
West Midlands	2389 (11.62)	8502 (11.91)
Yorkshire And The Humber	760 (3.70)	2757 (3.86)
Index of multiple deprivation – patient (%)^c^		
1 – least deprived	3057 (14.86)	13 637 (19.10)
2	3337 (16.22)	13 891 (19.46)
3	3877 (18.85)	13 973 (19.58)
4	4847 (23.57)	15 676 (21.96)
5 – most deprived	5450 (26.50)	14 204 (19.90)
BMI category at index^d^		
Underweight	758 (3.69)	1596 (2.24)
Normal range	11 992 (58.30)	42 004 (58.84)
Overweight	4510 (21.93)	17 279 (24.21)
Obese	3308 (16.08)	10 502 (14.71)
Smoking status at index^e^		
Current smoker	8937 (43.45)	20 750 (29.07)
Ex-smoker	3495 (16.99)	14 746 (20.66)
Never smoker	8136 (39.56)	35 885 (50.27)
Drug misuse at index	1440 (7.00)	742 (1.04)
Alcohol misuse at index	1618 (7.87)	1306 (1.83)
Physical health conditions at index		
None	11 579 (56.30)	43 480 (60.91)
One	5308 (25.81)	17 082 (23.93)
More than one	3681 (17.90)	10 819 (15.16)
Died (%)	2089 (10.16)	4534 (6.35)
Age at death [median (IQR)]	77.91 [62.89–87.03]	83.42 [73.06–89.97]
Follow up time [median (IQR)]	4.34 [2.30–7.94]	4.86 [2.55–8.66]
Baseline time [median (IQR)]	5.45 [0.91–15.18]	8.87 [2.86–18.63]

a Index is date of SMI diagnosis or, for patients without SMI, the diagnosis date of the matched patient with SMI.

b Ethnicity: missing coded as white [SMI: 7886 (38.34%); comparators: 30 922 (43.32%)].

c Index of multiple deprivation: missing patient IMD coded as primary care practice IMD [SMI: 44 (0.21%); comparators: 102 (0.14%)].

d BMI: missing coded as normal range [SMI: 5216 (25.36%); comparators: 18 178 (25.47%)].

e Smoking: missing coded as never smoker [SMI: 2467 (11.99%); comparators: 9233 (12.93%)].

### Demographic and clinical factors

A higher proportion of patients with SMI were identified as Black ethnic group (4.85% *v.* 3.00%) and from the most deprived IMD quintile (26.50% *v.* 19.90%) compared to comparators. Patients with SMI had a higher prevalence of physical health conditions and multimorbidity than comparators and were more likely to have a recording of obesity, current smoking, substance misuse recorded in their primary care record (Table 1) and used primary care more frequently in the year prior to and following SMI diagnosis than comparators (online Supplementary Table S6).

### Primary outcomes: admissions for physical health

**Fig. 1.**
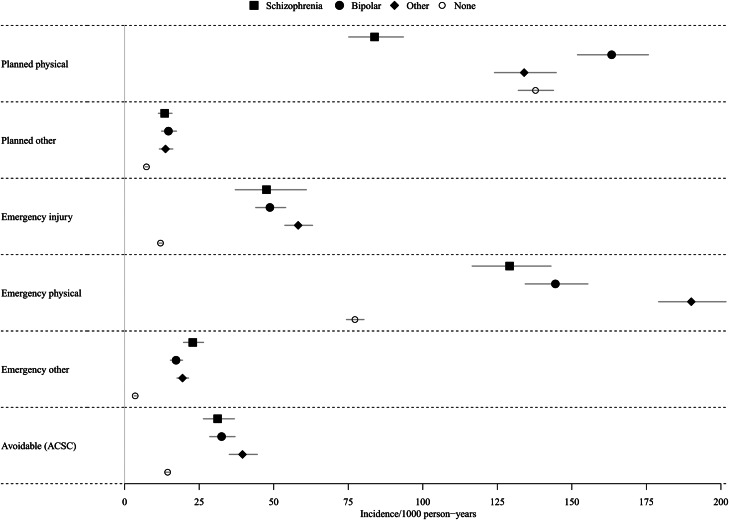
Unadjusted incidence of hospital admission outcomes per 1000-person years. Planned physical: Planned admissions for physical health. Planned other: Planned admissions for other non-mental health causes. Emergency injury: Emergency admissions for accidents, injuries and substance misuse. Emergency physical: All emergency physical admissions, including avoidable admissions. Emergency other: Emergency admissions for other non-mental health causes. Avoidable (ACSC): Potentially avoidable admissions, defined as ambulatory care-sensitive conditions.

**Fig. 2.**
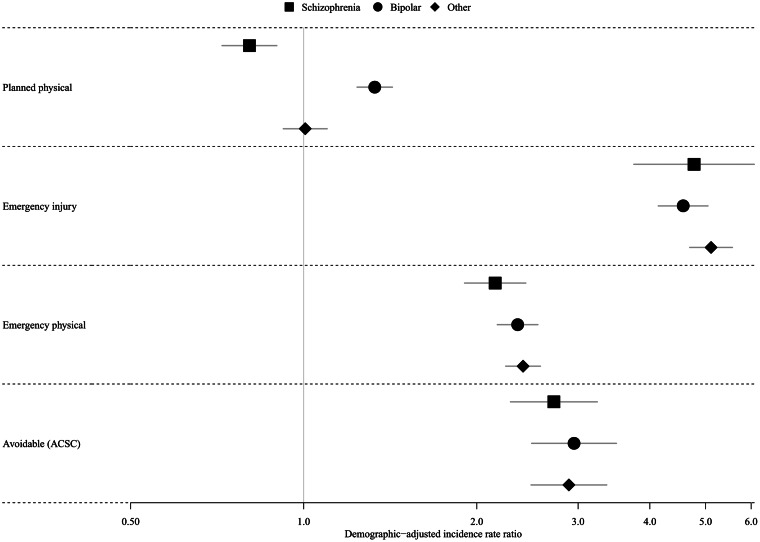
Demographically adjusted incidence rate ratios (IRR) of hospital admission outcomes in patients with SMI compared to comparators. Emergency injury: Emergency admissions for accidents, injuries and substance misuse. Emergency physical: All emergency physical admissions, including avoidable admissions. ACSC: Avoidable admissions, defined as ambulatory care-sensitive conditions.

The crude incidence of emergency and planned physical health admissions was higher than other non-mental health emergency causes, for patients with and without a diagnosis of SMI (Fig. 1, Table 2). Patients with a diagnosis of schizophrenia and other psychoses had more emergency physical health admissions than planned physical health admissions, whereas those with bipolar disorder or no SMI diagnosis had fewer (Fig. 1, Table 2). When stratified by SMI diagnosis, the crude incidence of emergency physical health admissions was elevated compared to comparators for all three SMI diagnostic groups (Fig. 1) and there was little difference in aIRRs across SMI groups (Table 2; Fig. 2). Controlling for physical health conditions increased the aIRR for those with schizophrenia while it decreased it for those with bipolar disorder and other psychoses (Table 2). When all SMI diagnoses were pooled, people with SMI had 2.33 times more admissions than people without SMI (aIRR: 2.33; 95% CI 2.22–2.46) after adjusting for demographic variables (age, sex, region, ethnicity, IMD, year of index). This effect remained even after controlling for health risk factors (BMI, smoking, alcohol, and drug misuse) and underlying physical health conditions (aIRR: 2.07; 95% CI 1.97–2.18, Table 2).

**Table 2.** Crude and adjusted incidence rate ratio of non-mental health hospital admissions in patients with SMI compared to matched comparators

		No SMI	Any SMI	Schizophrenia	Bipolar disorder	Other psychoses
	Person-years of follow up	427 101.69	114 427.02	27 148.23	43 111.44	44 167.35
Non-mental health admissions						
Planned physical health	Admissions, *n*	59 508	15 173	2199	7072	5902
	Crude incidence per 1000 (95% CI)	137.83 (132.07–143.85)	133.54 (126.68–140.78)	83.83 (75.19–93.48)	163.36 (151.91–175.67)	134.01 (124.06–144.75)
	Incidence rate ratio (95% CI)					
	Crude	1.00 (ref)	0.97 (0.91–1.03)	0.61 (0.54–0.68)	1.18 (1.10–1.28)	0.97 (0.89–1.06)
	Demographic adjusted	1.00 (ref)	1.08 (1.02–1.15)	0.80 (0.72–0.90)	1.33 (1.24–1.43)	1.01 (0.92–1.10)
	Demographic and physical health adjusted	1.00 (ref)	1.06 (1.00–1.12)	0.81 (0.73–0.90)	1.31 (1.22–1.40)	0.96 (0.88–1.05)
	Demographic and risk factor adjusted	1.00 (ref)	1.05 (0.99–1.12)	0.79 (0.70–0.88)	1.27 (1.18–1.37)	0.99 (0.91–1.08)
	Demographic, physical health and risk factor adjusted	1.00 (ref)	1.03 (0.98–1.09)	0.79 (0.71–0.88)	1.26 (1.17–1.35)	0.95 (0.87–1.04)
Emergency physical health	Admissions, *n*	29 797	15 555	2945	5704	6906
	Crude incidence	77.28 (74.42–80.24)	159.36 (152.05–167.01)	129.12 (116.6–142.99)	144.5 (134.38–155.37)	190.07 (179.13–201.68)
	Incidence rate ratio (95% CI)					
	Crude	1.00 (ref)	2.08 (2.00–2.17)	1.68 (1.52–1.85)	1.89 (1.76–2.03)	2.49 (2.34–2.64)
	Demographic adjusted	1.00 (ref)	2.33 (2.22–2.46)	2.15 (1.90––2.43)	2.35 (2.17–2.55)	2.41 (2.25–2.58)
	Demographic and physical health adjusted	1.00 (ref)	2.26 (2.15–2.38)	2.22 (1.96–2.51)	2.26 (2.09–2.43)	2.28 (2.14–2.44)
	Demographic and risk factor adjusted	1.00 (ref)	2.12 (2.02–2.24)	1.96 (1.75–2.20)	2.13 (1.96–2.32)	2.20 (2.06–2.35)
	Demographic, physical health and risk factor adjusted	1.00 (ref)	2.07 (1.97–2.18)	2.03 (1.80–2.28)	2.07 (1.91–2.23)	2.09 (1.96–2.23)
Emergency accidents, injuries and substance misuse	Admissions, *n*	4910	5423	1195	1949	2279
	Crude incidence	12.05 (11.49–12.63)	52.38 (48.39–56.7)	47.57 (37.14–60.93)	48.73 (43.99–53.99)	58.2 (53.76–63.01)
	Incidence rate ratio (95% CI)					
	Crude	1.00 (ref)	4.37 (4.05–4.72)	3.93 (3.05–5.05)	4.07 (3.67–4.53)	4.91 (4.51–5.35)
	Demographic adjusted	1.00 (ref)	4.84 (4.48–5.22)	4.77 (3.75–6.08)	4.57 (4.14–5.05)	5.11 (4.69–5.57)
	Demographic and physical health adjusted	1.00 (ref)	4.76 (4.41–5.14)	4.81 (3.77–6.15)	4.51 (4.09–4.98)	4.95 (4.56–5.38)
	Demographic and risk factor adjusted	1.00 (ref)	4.11 (3.80–4.45)	4.06 (3.11–5.29)	4.03 (3.64–4.46)	4.22 (3.87–4.60)
	Demographic, physical health and risk factor adjusted	1.00 (ref)	4.07 (3.75–4.41)	4.12 (3.13–5.42)	4.00 (3.62–4.42)	4.10 (3.77–4.46)
Avoidable admissions for ambulatory care sensitive conditions (ACSC) compared to emergency admissions for non-ACSC physical health conditions						
All ACSC	Admissions, *n*	5596	3505	688	1331	1486
	Crude incidence	14.42 (13.66–15.23)	35.01 (32.4–37.84)	31.19 (26.44–36.78)	32.52 (28.57–37.01)	39.54 (35.13–44.49)
	Incidence rate ratio (95% CI)					
	Crude	1.00 (ref)	2.47 (2.26–2.69)	2.18 (1.84–2.59)	2.28 (2.00–2.60)	2.80 (2.47–3.18)
	Demographic adjusted	1.00 (ref)	2.88 (2.60–3.19)	2.72 (2.29–3.24)	2.95 (2.49–3.50)	2.89 (2.49–3.37)
	Demographic and physical health adjusted	1.00 (ref)	2.82 (2.55–3.11)	2.94 (2.51–3.44)	2.79 (2.35–3.31)	2.78 (2.43–3.18)
	Demographic and risk factor adjusted	1.00 (ref)	2.59 (2.33–2.88)	2.48 (2.08–2.97)	2.69 (2.26–3.20)	2.56 (2.22–2.96)
	Demographic, physical health and risk factor adjusted	1.00 (ref)	2.55 (2.30–2.82)	2.65 (2.25–3.12)	2.57 (2.16–3.06)	2.48 (2.17–2.83)
	ACSC adjusted	1.00 (ref)	2.39 (2.17–2.64)	2.47 (2.12–2.89)	2.43 (2.06–2.87)	2.31 (2.05–2.62)
Non-ACSC emergency physical health	Admissions, *n*	24 299	12 105	2266	4389	5450
	Crude incidence	62.3 (59.95–64.74)	122.57 (116.75–128.68)	96.15 (86.07–107.42)	111.01 (103.33–119.27)	148.35 (139.53–157.74)
	Incidence rate ratio (95% CI)					
	Crude	1.00 (ref)	1.99 (1.90–2.08)	1.55 (1.40–1.72)	1.80 (1.68–1.93)	2.41 (2.26–2.56)
	Demographic adjusted	1.00 (ref)	2.19 (2.07–2.31)	2.00 (1.74–2.30)	2.21 (2.03–2.40)	2.26 (2.11–2.43)
	Demographic and physical health adjusted	1.00 (ref)	2.12 (2.01–2.24)	2.04 (1.77–2.35)	2.13 (1.98–2.31)	2.16 (2.01–2.32)
	Demographic and risk factor adjusted	1.00 (ref)	2.00 (1.90–2.11)	1.84 (1.62–2.08)	2.00 (1.84–2.17)	2.09 (1.96–2.24)
	Demographic, physical health and risk factor adjusted	1.00 (ref)	1.92 (1.82–2.03)	1.88 (1.65–2.14)	1.96 (1.81–2.11)	2.00 (1.87–2.14)
	ACSC adjusted	1.00 (ref)	1.92 (1.82–2.02)	1.85 (1.62–2.11)	1.92 (1.78–2.08)	1.96 (1.83–2.09)
Avoidable admissions for ambulatory care sensitive conditions (ACSC) by ACSC category						
Chronic ACSC	Admissions, *n*	3367	2273	447	867	959
	Crude incidence	8.83 (8.25–9.46)	23.06 (20.67–25.73)	20.85 (16.69–26.03)	21.25 (17.87–25.28)	26.06 (21.89–31.02)
	Incidence rate ratio (95% CI)					
	Crude	1.00 (ref)	2.65 (2.35–3.00)	2.38 (1.89–3.00)	2.44 (2.03–2.93)	3.01 (2.50–3.62)
	Demographic adjusted	1.00 (ref)	3.46 (2.97–4.02)	3.22 (2.53–4.09)	3.48 (2.71–4.46)	3.56 (2.85–4.45)
	ACSC adjusted	1.00 (ref)	2.72 (2.34–3.16)	2.82 (2.28–3.49)	2.85 (2.22–3.67)	2.55 (2.13–3.07)
Acute ACSC	Admissions, *n*	1319	722	144	295	283
	Crude incidence	3.18 (2.96–3.41)	6.66 (6.09–7.28)	5.82 (4.7–7.19)	7.12 (6.11–8.28)	6.65 (5.85–7.56)
	Incidence rate ratio (95% CI)					
	Crude	1.00 (ref)	2.11 (1.90–2.34)	1.85 (1.47–2.31)	2.24 (1.92–2.61)	2.13 (1.84–2.47)
	Demographic adjusted	1.00 (ref)	2.13 (1.91–2.37)	2.10 (1.66–2.65)	2.31 (1.98–2.69)	1.98 (1.71–2.29)
	ACSC adjusted	1.00 (ref)	1.91 (1.71–2.14)	1.91 (1.51–2.40)	2.04 (1.74–2.39)	1.79 (1.54–2.09)
Vaccine preventable ACSC	Admissions, *n*	910	510	97	169	244
	Crude incidence	2.23 (2.05–2.42)	4.79 (4.32–5.31)	3.94 (3.08–5.03)	4 (3.37–4.74)	6.17 (5.3–7.18)
	Incidence rate ratio (95% CI)					
	Crude	1.00 (ref)	2.20 (1.96–2.47)	1.78 (1.38–2.29)	1.82 (1.52–2.18)	2.83 (2.41–3.31)
	Demographic adjusted	1.00 (ref)	2.33 (2.08–2.62)	2.33 (1.81–2.99)	2.27 (1.89–2.72)	2.38 (2.03–2.80)
	ACSC adjusted	1.00 (ref)	2.08 (1.85–2.34)	2.26 (1.75–2.92)	1.99 (1.67–2.38)	2.07 (1.78–2.42)

Demographic adjusted: for age at study entry, sex, IMD, ethnicity, region, year of diagnosis.

Risk factor adjusted: BMI, smoking, drug misuse, alcohol misuse.

Physical health adjusted: 24 physical health conditions.

ACSC adjusted: Adjusted for demographic variables, plus risk factors, individual ACSC chronic conditions and a count of non-ACSC physical health conditions.

In contrast, the incidence of planned admissions differed by SMI diagnosis. Patients with schizophrenia had fewer planned admissions compared to comparators (aIRR: 0.80; 95% CI 0.72–0.90), while those with bipolar disorder had more (aIRR: 1.33; 95% CI 1.24–1.43; Table 2 & Fig. 2). As with the emergency physical health admissions, controlling for health risk factors (BMI, smoking, alcohol, and drug misuse) and underlying physical health conditions did not alter the findings, and controlling for physical health decreased the aIRR for patients with bipolar disorder and other psychoses; but increased the aIRR for patients with schizophrenia.

The *E* value for the fully adjusted IRR of planned physical admissions in people with schizophrenia compared to those without SMI was 1.85, suggesting an unmeasured confounder would need an effect size of 1.85 in relation to both schizophrenia and hospitalisation to confound this finding. For the IRR of emergency physical health admissions in those with SMI *v.* no SMI, the *E* value was 3.56.

### Secondary outcomes: avoidable admissions and emergency accident, injury, and substance use admissions

The incidence of emergency avoidable admissions compared to those without SMI was similar ion patients with schizophrenia, bipolar disorder and other psychoses (Table 2; Fig. 2). When pooled across diagnoses, patients with SMI had a higher incidence of emergency avoidable admissions than those without SMI (aIRR: 2.88; 95% CI 2.60–3.19). This remained, even after controlling for prior diagnosis of individual physical health conditions included in the ACSC definition and a count of other physical health conditions (aIRR: 2.39; 95% CI 2.17–2.64); and was higher than for emergency physical health admissions not defined as avoidable (Table 2). When stratified by type of avoidable admission, the aIRR was highest for admissions due to chronic physical health conditions (aIRR: 3.46; 95% CI 2.97–4.02; Table 2).

Rates of emergency accident, injury and substance misuse admissions were lower than emergency physical health admissions (Fig. 1), however patients with an SMI had markedly elevated rates of emergency accident, injury and substance misuse admissions compared to the non-SMI comparator population (aIRR: 4.84; 95% CI 4.48–5.22).

The *E* value for the fully adjusted IRR of emergency avoidable admissions in those with *v.* without SMI was 4.21, and for admissions for accidents, injuries and substance misuse admissions was 7.60

### Sensitivity analyses

Limiting the outcome period to the first year following diagnosis, increased the crude and adjusted IRR for all outcomes, particularly for emergency admissions for accidents, injuries, and substance misuse (sensitivity: aIRR: 6.18; 95% CI 5.46–6.98 *v.* main model: 4.84; 95% CI 4.48–5.22, online Supplementary Table S7).

Controlling for the number of primary care consultations and emergency and planned non-mental health admissions in the year prior to index attenuated our main findings for all outcomes; the incidence of emergency physical (aIRR: 1.52; 95% CI 1.44–1.59), avoidable admissions (aIRR:1.85, 95% CI 1.67–2.04) and emergency accident, injury, and substance misuse admissions (aIRR: 3.06; 95% CI 2.87–3.27) remained elevated (online Supplementary Table S8). Excluding those patients with less than one year's baseline data (online Supplementary Table S8) and updating physical health and risk factor covariates to account for better capture in the year following diagnosis (online Supplementary Table S9) or controlling for individual physical health conditions (online Supplementary Table S10) did not alter the findings. Using zero-inflated negative binomial regression resulted in a lower aIRR for emergency and planned physical health admissions, though the effect sizes had overlapping confidence intervals with those of the negative binomial model, and it did not alter the findings (online Supplementary Table S11).

## Discussion

Physical health admissions in NHS secondary care were the most frequent non-mental health reason for hospital admissions for those with and without SMI. People with SMI were over twice as likely to have an emergency physical health admission during the study period than those without SMI, and the rate of admissions was similar across SMI diagnoses. This remained true after controlling for a wide range of demographic and clinical factors.

Emergency admissions due to chronic avoidable conditions were almost three and a half times the admission rate in patients with SMI compared to those without SMI. Given the high prevalence of diabetes, COPD and neurological disease in people with SMI (Launders et al., 2022), this represents a major burden on the NHS. Controlling for pre-existing physical health conditions did not alter our findings, suggesting that the number of underlying physical health conditions that a patient has recorded in primary care is not the only driver of the high rate of physical health admissions in people with SMI. In contrast, the high rate of avoidable admissions for chronic conditions suggests that severity or control of pre-existing physical health conditions leads to high emergency admissions for physical health. As these conditions are thought to be preventable or can be reduced by actions in primary care or community settings, this suggests that targeted approaches to improving physical health screening, and the monitoring and treatment of chronic physical health conditions in community settings are needed to reduce emergency hospital admissions in this population. While physical health checks are incentivised in primary care in the UK, a previous study found that while care plans reduced avoidable admissions in those with SMI, physical health checks for BMI, blood pressure, cholesterol and glucose did not (Ride et al., 2018). A range of interventions are therefore likely required to reduce avoidable admissions in those with SMI, such as physical health clinics in psychiatric services, more thorough physical health checks and screening, improved uptake of care plans, and personalised care and support to better manage existing physical health conditions.

Furthermore, people with SMI also have a high prevalence of modifiable health risk factors (Launders et al., 2022), which may increase the severity or affect the management of physical health conditions. Interventions aimed at reducing these, such as smoking or alcohol cessation services have been shown to be effective (Gilbody et al., 2019; McDonell et al., 2017), and may also act to reduce emergency admissions.

The year following SMI diagnosis appears to be a time of high emergency hospital admissions utilisation in people with SMI. We found that people with SMI were over five times as likely to be admitted for accidents, injuries and substance misuse, and almost three times as likely to have an avoidable admission in the year following SMI diagnosis. This suggests that targeted interventions around the time of diagnosis are required, particularly focusing on risk reduction and management of existing physical health conditions.

Excess emergency admissions reduce the quality and length of life for those with SMI and represent a cost burden to the NHS. Using a prevalence of SMI in 2015/2016 of 0.89% (Grigoroglou et al., 2020), and a population of 43.8 million people aged 18 or over in 2017 in England; this suggests an excess of between 35 000 to 45 000 admissions per year, likely to cost in excess of £55 million (using an average emergency admission cost of £1603 in 2017/2018) (NHS Improvement, 2019).

In contrast to the findings of all emergency admission outcomes, there were marked differences by SMI diagnosis for planned physical health admissions, and we found that pooling results for all SMI diagnoses potentially masks differences in planned healthcare utilisation. People with bipolar disorder had elevated rates of planned physical health admissions, while those with schizophrenia had far lower rates. While low rates of emergency inpatient admissions are usually deemed to be a sign of good preventative and primary care, the lower-than-expected rates of planned admissions for physical health in people with schizophrenia are of concern, and suggest potential barriers to access of planned inpatient care in this population. However, while there has been research into barriers to attending screening and other disease-specific specialist services (Bramberg, Torgerson, Kjellstrom, Welin, & Rusner, 2018; Melamed et al., 2019; Murphy et al., 2021), as well as primary care (Bramberg et al., 2018; Decoux, 2005; Kohn et al., 2021; Mitchell et al., 2022), there is less information on barriers to planned inpatient admissions. We need to understand the reasons for reduced rates of planned care specifically in people with schizophrenia as this may contribute to the mortality gap for this group of patients.

### Strengths and limitations

To our knowledge, this is the first study to investigate both planned and emergency admissions in people with SMI, and the first longitudinal study based in England investigating admissions in people with SMI compared to matched comparators. While previous studies have found high rates of potentially avoidable admissions in those with SMI (Davydow et al., 2016; Lin et al., 2011; Mai, Holman, Sanfilippo, & Emery, 2011), the size of this cohort allows for stratification by SMI diagnosis and investigation of causes other than avoidable ACSC admissions, furthering the understanding of the differences in hospital utilisation in people with SMI.

The use of linked primary and secondary care records is a major strength of our study. Many studies of hospital admissions use only hospital data and are therefore limited to patients who attend hospital during a baseline period. The longitudinal nature of primary care records in England, and richness of data they contain, allows for a more complete capture of demographic and clinical data and a more accurate comparison of hospital admission rates between those with and without SMI in the general population.

We were able to control for known causes of variation in hospital admissions at the patient level, such as age, sex, ethnicity and deprivation (Busby, Purdy, & Hollingworth, 2017a). Patients with SMI were matched with comparators in the same primary care practice, reducing the known variation in hospital admission rates between practices, and regionally in England (Busby et al., 2017b). Controlling for pre-existing physical health conditions did not alter our findings, suggesting that the number of underlying physical health conditions that a patient has recorded in primary care is not the only driver of the high rate of physical health admissions in people with SMI. This is supported by a previous study of avoidable ACSC conditions in those with SMI (Davydow et al., 2016). For those with schizophrenia, controlling for pre-existing physical health conditions did not reduce the IRR of any of the hospital outcomes, possibly due to an under-ascertainment of physical disease in this population (Gabilondo, Alonso-Moran, Nuno-Solinis, Orueta, & Iruin, 2017; Launders et al., 2022; Smith, Langan, McLean, Guthrie, & Mercer, 2013).

While we controlled for physical health conditions, we did not control for other mental health diagnoses. It is recognised that physical health admissions are elevated in people with anxiety and depression (Guthrie et al., 2016) and high rates of these diagnoses in those with SMI may therefore contribute to high admissions. However, whether depression and anxiety is a symptom of SMI or a separate condition may not be clear (Upthegrove, Marwaha, & Birchwood, 2017), and therefore the role of other mental health diagnoses on hospitalisations in this population is hard to determine.”

While we were able to control for a range of covariates, the matching strategy of this cohort could have introduced bias. People with SMI were matched to those who did not develop SMI at any time prior to or during follow up. Given those with SMI are known to use more healthcare resources in the years prior to SMI diagnosis (Wallace et al., 2020), this could have resulted in the under-estimation of hospital utilisation in the comparator population. However, given that the incidence of SMI is low (Hardoon et al., 2013) the probability of matching a person with SMI to a person who will develop SMI in the future is also low, and therefore the impact is probably limited.

In the general population, hospital use in the year prior to outcome period may be the strongest predictor of hospital admissions (Donnan, Dorward, Mutch, & Morris, 2008; Hippisley-Cox & Coupland, 2013), and therefore useful for predicting which individuals will use hospital services. However, in models comparing admissions between groups it may represent over-adjustment, since the same causal factors driving admissions may be present prior to and after index date, particularly in the year prior to SMI diagnosis, where healthcare use may be elevated (Wallace et al., 2020). In our study, including these variables attenuated all effect sizes but did not alter the main findings.

While CPRD is broadly representative of England's population (Herrett et al., 2015; Wolf et al., 2019), there may be residual confounding due to missing data or linkage bias. In particular, the capture of physical health diagnoses in primary care may be subject to surveillance bias, whereby those who attend primary care more often have more opportunities to have physical health conditions diagnosed and recorded.

Finally, we did not investigate the length of stay in this study. Previous research has found that people with SMI have longer non-mental health hospital stays than those without SMI (Ronaldson et al., 2020) and higher hospital costs for avoidable admissions (Sporinova et al., 2019). These periods of admission and stays in inpatient psychiatric care represent periods of time where the patient is unable to experience admissions and could therefore have lowered the admission rate in those with SMI.

While the average length of stay is elevated, those with SMI may also be at increased risk of discharge against medical advice. While we could find no studies investigating discharge disposition for physical health admissions in people with SMI, this could result in a greater risk of multiple short admissions, and drive some of the high 30-readmission rates observed in this population (Germack et al., 2018; Ronaldson et al., 2020). The likely complex pattern of the length of stays for physical health admissions, reasons for discharge, and the impact this has on hospital utilisation in those with SMI requires further research.

## Conclusions

We found that people with SMI have a markedly increased rate of emergency physical health admissions compared to people without SMI. We also identified an under-utilisation of planned physical health hospital services in those with schizophrenia. Further research is required to determine the drivers of high-frequency admissions in those with underlying physical disease, and potential barriers to optimal use of healthcare services in this population. To reduce physical health admissions in people with SMI, targeted interventions are required in community and psychiatric services, aimed at improving access to appropriate care, ensuring effective physical health screening, and providing support to enable appropriate treatment and control of chronic physical health conditions.

## Supplementary material

For supplementary material accompanying this paper visit https://doi.org/10.1017/S0033291722002811. click here to view supplementary material
