# Supplementary material for: The incidence rate of planned and emergency physical health hospital admissions in people diagnosed with severe mental illness: a cohort study
Source: Psychol Med. 2022 Sep 7;53(12):5603–14. doi: 10.1017/S0033291722002811 (PMC10482715; doi:10.1017/S0033291722002811)
Supplement: Supplementary file 1 [file S0033291722002811sup001.docx]

# Supplementary Table 1: Read code list to define SMI

| **Read code** | **Term** | **Group** |
| --- | --- | --- |
| E110 | Manic disorder, single episode | bipolar |
| E110-1 | Hypomanic psychoses | bipolar |
| E110-99 | Mania/hypomania | bipolar |
| E1100 | Single manic episode, unspecified | bipolar |
| E1101 | Single manic episode, mild | bipolar |
| E1102 | Single manic episode, moderate | bipolar |
| E1103 | Single manic episode, severe without mention of psychosis | bipolar |
| E1104 | Single manic episode, severe, with psychosis | bipolar |
| E1105 | Single manic episode in partial or unspecified remission | bipolar |
| E1106 | Single manic episode in full remission | bipolar |
| E110z | Manic disorder, single episode NOS | bipolar |
| E111 | Recurrent manic episodes | bipolar |
| E1110 | Recurrent manic episodes, unspecified | bipolar |
| E1111 | Recurrent manic episodes, mild | bipolar |
| E1112 | Recurrent manic episodes, moderate | bipolar |
| E1113 | Recurrent manic episodes, severe without mention psychosis | bipolar |
| E1114 | Recurrent manic episodes, severe, with psychosis | bipolar |
| E1115 | Recurrent manic episodes, partial or unspecified remission | bipolar |
| E1116 | Recurrent manic episodes, in full remission | bipolar |
| E111z | Recurrent manic episode NOS | bipolar |
| E114 | Bipolar affective disorder, currently manic | bipolar |
| E114-1 | Manic-depressive - now manic | bipolar |
| E1140 | Bipolar affective disorder, currently manic, unspecified | bipolar |
| E1141 | Bipolar affective disorder, currently manic, mild | bipolar |
| E1142 | Bipolar affective disorder, currently manic, moderate | bipolar |
| E1143 | Bipolar affect disord, currently manic, severe, no psychosis | bipolar |
| E1144 | Bipolar affect disord, currently manic,severe with psychosis | bipolar |
| E1145 | Bipolar affect disord,currently manic, part/unspec remission | bipolar |
| E1146 | Bipolar affective disorder, currently manic, full remission | bipolar |
| E114z | Bipolar affective disorder, currently manic, NOS | bipolar |
| E115 | Bipolar affective disorder, currently depressed | bipolar |
| E115-1 | Manic-depressive - now depressed | bipolar |
| E1150 | Bipolar affective disorder, currently depressed, unspecified | bipolar |
| E1151 | Bipolar affective disorder, currently depressed, mild | bipolar |
| E1152 | Bipolar affective disorder, currently depressed, moderate | bipolar |
| E1153 | Bipolar affect disord, now depressed, severe, no psychosis | bipolar |
| E1154 | Bipolar affect disord, now depressed, severe with psychosis | bipolar |
| E1155 | Bipolar affect disord, now depressed, part/unspec remission | bipolar |
| E1156 | Bipolar affective disorder, now depressed, in full remission | bipolar |
| E115z | Bipolar affective disorder, currently depressed, NOS | bipolar |
| E116 | Mixed bipolar affective disorder | bipolar |
| E1160 | Mixed bipolar affective disorder, unspecified | bipolar |
| E1161 | Mixed bipolar affective disorder, mild | bipolar |
| E1162 | Mixed bipolar affective disorder, moderate | bipolar |
| E1163 | Mixed bipolar affective disorder, severe, without psychosis | bipolar |
| E1164 | Mixed bipolar affective disorder, severe, with psychosis | bipolar |
| E1165 | Mixed bipolar affective disorder, partial/unspec remission | bipolar |
| E1166 | Mixed bipolar affective disorder, in full remission | bipolar |
| E116z | Mixed bipolar affective disorder, NOS | bipolar |
| E117 | Unspecified bipolar affective disorder | bipolar |
| E1170 | Unspecified bipolar affective disorder, unspecified | bipolar |
| E1171 | Unspecified bipolar affective disorder, mild | bipolar |
| E1172 | Unspecified bipolar affective disorder, moderate | bipolar |
| E1173 | Unspecified bipolar affective disorder, severe, no psychosis | bipolar |
| E1174 | Unspecified bipolar affective disorder,severe with psychosis | bipolar |
| E1175 | Unspecified bipolar affect disord, partial/unspec remission | bipolar |
| E1176 | Unspecified bipolar affective disorder, in full remission | bipolar |
| E117z | Unspecified bipolar affective disorder, NOS | bipolar |
| E11y | Other and unspecified manic-depressive psychoses | bipolar |
| E11y0 | Unspecified manic-depressive psychoses | bipolar |
| E11y1 | Atypical manic disorder | bipolar |
| E11y3 | Other mixed manic-depressive psychoses | bipolar |
| E11yz | Other and unspecified manic-depressive psychoses NOS | bipolar |
| E11z | Other and unspecified affective psychoses | bipolar |
| E11z0 | Unspecified affective psychoses NOS | bipolar |
| E11zz | Other affective psychosis NOS | bipolar |
| Eu30 | [X]Manic episode | bipolar |
| Eu30-1 | [X]Bipolar disorder, single manic episode | bipolar |
| Eu300 | [X]Hypomania | bipolar |
| Eu301 | [X]Mania without psychotic symptoms | bipolar |
| Eu302 | [X]Mania with psychotic symptoms | bipolar |
| Eu302-1 | [X]Mania with mood-congruent psychotic symptoms | bipolar |
| Eu302-2 | [X]Mania with mood-incongruent psychotic symptoms | bipolar |
| Eu302-3 | [X]Manic stupor | bipolar |
| Eu30y | [X]Other manic episodes | bipolar |
| Eu30z | [X]Manic episode, unspecified | bipolar |
| Eu30z-1 | [X]Mania NOS | bipolar |
| Eu31 | [X]Bipolar affective disorder | bipolar |
| Eu31-1 | [X]Manic-depressive illness | bipolar |
| Eu31-2 | [X]Manic-depressive psychosis | bipolar |
| Eu31-3 | [X]Manic-depressive reaction | bipolar |
| Eu310 | [X]Bipolar affective disorder, current episode hypomanic | bipolar |
| Eu311 | [X]Bipolar affect disorder cur epi manic wout psychotic symp | bipolar |
| Eu312 | [X]Bipolar affect disorder cur epi manic with psychotic symp | bipolar |
| Eu313 | [X]Bipolar affect disorder cur epi mild or moderate depressn | bipolar |
| Eu314 | [X]Bipol aff disord, curr epis sev depress, no psychot symp | bipolar |
| Eu315 | [X]Bipolar affect dis cur epi severe depres with psyc symp | bipolar |
| Eu316 | [X]Bipolar affective disorder, current episode mixed | bipolar |
| Eu317 | [X]Bipolar affective disorder, currently in remission | bipolar |
| Eu318 | [X]Bipolar affective disorder type I | bipolar |
| Eu31y | [X]Other bipolar affective disorders | bipolar |
| Eu31y-1 | [X]Bipolar II disorder | bipolar |
| Eu31y-2 | [X]Recurrent manic episodes | bipolar |
| Eu31z | [X]Bipolar affective disorder, unspecified | bipolar |
| Eu333-2 | [X]Manic-depress psychosis,depressed type+psychotic symptoms | bipolar |
| E11 | Affective psychoses | bipolar |
| E11-1 | Bipolar psychoses | bipolar |
| E11-99 | Manic-depressive psychoses | bipolar |
| Eu3z-1 | [X]Affective psychosis NOS | bipolar |
| 146D | H/O: manic depressive disorder | bipolar |
| 1S42 | Manic mood | bipolar |
| ZV111-1 | [V]Personal history of manic-depressive psychosis | bipolar |
| ZV111-2 | [V]Personal history of manic-depressive psychosis | bipolar |
| Eu332-3 | [X]Manic-depress psychosis,depressd,no psychotic symptoms | bipolar |
| E104-1 | Oneirophrenia | other psychosis |
| E105 | Latent schizophrenia | other psychosis |
| E1050 | Unspecified latent schizophrenia | other psychosis |
| E1051 | Subchronic latent schizophrenia | other psychosis |
| E1052 | Chronic latent schizophrenia | other psychosis |
| E1053 | Acute exacerbation of subchronic latent schizophrenia | other psychosis |
| E1054 | Acute exacerbation of chronic latent schizophrenia | other psychosis |
| E1055 | Latent schizophrenia in remission | other psychosis |
| E105z | Latent schizophrenia NOS | other psychosis |
| E107-1 | Cyclic schizophrenia | other psychosis |
| E107-99 | Acute schizo affective psychosis | other psychosis |
| E12 | Paranoid states | other psychosis |
| E12-99 | Paranoia | other psychosis |
| E120 | Simple paranoid state | other psychosis |
| E121 | Chronic paranoid psychosis | other psychosis |
| E121-1 | Sander's disease | other psychosis |
| E122 | Paraphrenia | other psychosis |
| E123 | Shared paranoid disorder | other psychosis |
| E123-1 | Folie a deux | other psychosis |
| E12y | Other paranoid states | other psychosis |
| E12y0 | Paranoia querulans | other psychosis |
| E12yz | Other paranoid states NOS | other psychosis |
| E12z | Paranoid psychosis NOS | other psychosis |
| E13 | Other nonorganic psychoses | other psychosis |
| E13-1 | Reactive psychoses | other psychosis |
| E131 | Acute hysterical psychosis | other psychosis |
| E133 | Acute paranoid reaction | other psychosis |
| E133-1 | Bouffee delirante | other psychosis |
| E134 | Psychogenic paranoid psychosis | other psychosis |
| E13y | Other reactive psychoses | other psychosis |
| E13y0 | Psychogenic stupor | other psychosis |
| E13y1 | Brief reactive psychosis | other psychosis |
| E13yz | Other reactive psychoses NOS | other psychosis |
| E13z | Nonorganic psychosis NOS | other psychosis |
| E13z-1 | Psychotic episode NOS | other psychosis |
| Eu2 | [X]Schizophrenia, schizotypal and delusional disorders | other psychosis |
| Eu21 | [X]Schizotypal disorder | other psychosis |
| Eu21-1 | [X]Latent schizophrenic reaction | other psychosis |
| Eu21-2 | [X]Borderline schizophrenia | other psychosis |
| Eu21-3 | [X]Latent schizophrenia | other psychosis |
| Eu21-4 | [X]Prepsychotic schizophrenia | other psychosis |
| Eu21-5 | [X]Prodromal schizophrenia | other psychosis |
| Eu21-6 | [X]Pseudoneurotic schizophrenia | other psychosis |
| Eu21-7 | [X]Pseudopsychopathic schizophrenia | other psychosis |
| Eu21-8 | [X]Schizotypal personality disorder | other psychosis |
| Eu22 | [X]Persistent delusional disorders | other psychosis |
| Eu220 | [X]Delusional disorder | other psychosis |
| Eu220-1 | [X]Paranoid psychosis | other psychosis |
| Eu220-2 | [X]Paranoid state | other psychosis |
| Eu220-3 | [X]Paraphrenia - late | other psychosis |
| Eu220-4 | [X]Sensitiver Beziehungswahn | other psychosis |
| Eu220-5 | [X]Paranoia | other psychosis |
| Eu221 | [X]Delusional misidentification syndrome | other psychosis |
| Eu221-1 | [X]Capgras syndrome | other psychosis |
| Eu22y | [X]Other persistent delusional disorders | other psychosis |
| Eu22y-1 | [X]Delusional dysmorphophobia | other psychosis |
| Eu22y-2 | [X]Involutional paranoid state | other psychosis |
| Eu22y-3 | [X]Paranoia querulans | other psychosis |
| Eu22z | [X]Persistent delusional disorder, unspecified | other psychosis |
| Eu23 | [X]Acute and transient psychotic disorders | other psychosis |
| Eu230 | [X]Acute polymorphic psychot disord without symp of schizoph | other psychosis |
| Eu230-1 | [X]Bouffee delirante | other psychosis |
| Eu230-2 | [X]Cycloid psychosis | other psychosis |
| Eu231 | [X]Acute polymorphic psychot disord with symp of schizophren | other psychosis |
| Eu231-1 | [X]Bouffee delirante with symptoms of schizophrenia | other psychosis |
| Eu231-2 | [X]Cycloid psychosis with symptoms of schizophrenia | other psychosis |
| Eu232 | [X]Acute schizophrenia-like psychotic disorder | other psychosis |
| Eu232-1 | [X]Brief schizophreniform disorder | other psychosis |
| Eu232-2 | [X]Brief schizophrenifrm psych | other psychosis |
| Eu232-3 | [X]Oneirophrenia | other psychosis |
| Eu233 | [X]Other acute predominantly delusional psychotic disorders | other psychosis |
| Eu233-2 | [X]Psychogenic paranoid psychosis | other psychosis |
| Eu23y | [X]Other acute and transient psychotic disorders | other psychosis |
| Eu23z | [X]Acute and transient psychotic disorder, unspecified | other psychosis |
| Eu23z-1 | [X]Brief reactive psychosis NOS | other psychosis |
| Eu23z-2 | [X]Reactive psychosis | other psychosis |
| Eu24 | [X]Induced delusional disorder | other psychosis |
| Eu24-1 | [X]Folie a deux | other psychosis |
| Eu24-2 | [X]Induced paranoid disorder | other psychosis |
| Eu24-3 | [X]Induced psychotic disorder | other psychosis |
| Eu25 | [X]Schizoaffective disorders | other psychosis |
| Eu250 | [X]Schizoaffective disorder, manic type | other psychosis |
| Eu250-1 | [X]Schizoaffective psychosis, manic type | other psychosis |
| Eu250-2 | [X]Schizophreniform psychosis, manic type | other psychosis |
| Eu251 | [X]Schizoaffective disorder, depressive type | other psychosis |
| Eu251-1 | [X]Schizoaffective psychosis, depressive type | other psychosis |
| Eu251-2 | [X]Schizophreniform psychosis, depressive type | other psychosis |
| Eu252 | [X]Schizoaffective disorder, mixed type | other psychosis |
| Eu252-1 | [X]Cyclic schizophrenia | other psychosis |
| Eu252-2 | [X]Mixed schizophrenic and affective psychosis | other psychosis |
| Eu25y | [X]Other schizoaffective disorders | other psychosis |
| Eu25z | [X]Schizoaffective disorder, unspecified | other psychosis |
| Eu25z-1 | [X]Schizoaffective psychosis NOS | other psychosis |
| Eu2y | [X]Other nonorganic psychotic disorders | other psychosis |
| Eu2y-1 | [X]Chronic hallucinatory psychosis | other psychosis |
| Eu2z | [X]Unspecified nonorganic psychosis | other psychosis |
| Eu2z-1 | [X]Psychosis NOS | other psychosis |
| E11-3 | Manic psychoses | other psychosis |
| E1z | Non-organic psychosis NOS | other psychosis |
| 146H | H/O: psychosis | other psychosis |
| E1 | Non-organic psychoses | other psychosis |
| E1y | Other specified non-organic psychoses | other psychosis |
| Eu531-1 | [X]Puerperal psychosis NOS | other psychosis |
| Eu843-4 | [X]Symbiotic psychosis | other psychosis |
| E10 | Schizophrenic disorders | schizophrenia |
| E10-98 | Schizophrenic psychoses NOS | schizophrenia |
| E10-99 | Schizophrenic psychoses | schizophrenia |
| E100 | Simple schizophrenia | schizophrenia |
| E100-1 | Schizophrenia simplex | schizophrenia |
| E1000 | Unspecified schizophrenia | schizophrenia |
| E1001 | Subchronic schizophrenia | schizophrenia |
| E1002 | Chronic schizophrenic | schizophrenia |
| E1003 | Acute exacerbation of subchronic schizophrenia | schizophrenia |
| E1004 | Acute exacerbation of chronic schizophrenia | schizophrenia |
| E1005 | Schizophrenia in remission | schizophrenia |
| E100z | Simple schizophrenia NOS | schizophrenia |
| E101 | Hebephrenic schizophrenia | schizophrenia |
| E1010 | Unspecified hebephrenic schizophrenia | schizophrenia |
| E1011 | Subchronic hebephrenic schizophrenia | schizophrenia |
| E1012 | Chronic hebephrenic schizophrenia | schizophrenia |
| E1013 | Acute exacerbation of subchronic hebephrenic schizophrenia | schizophrenia |
| E1014 | Acute exacerbation of chronic hebephrenic schizophrenia | schizophrenia |
| E1015 | Hebephrenic schizophrenia in remission | schizophrenia |
| E101z | Hebephrenic schizophrenia NOS | schizophrenia |
| E102 | Catatonic schizophrenia | schizophrenia |
| E1020 | Unspecified catatonic schizophrenia | schizophrenia |
| E1021 | Subchronic catatonic schizophrenia | schizophrenia |
| E1022 | Chronic catatonic schizophrenia | schizophrenia |
| E1023 | Acute exacerbation of subchronic catatonic schizophrenia | schizophrenia |
| E1024 | Acute exacerbation of chronic catatonic schizophrenia | schizophrenia |
| E1025 | Catatonic schizophrenia in remission | schizophrenia |
| E102z | Catatonic schizophrenia NOS | schizophrenia |
| E103 | Paranoid schizophrenia | schizophrenia |
| E1030 | Unspecified paranoid schizophrenia | schizophrenia |
| E1031 | Subchronic paranoid schizophrenia | schizophrenia |
| E1032 | Chronic paranoid schizophrenia | schizophrenia |
| E1033 | Acute exacerbation of subchronic paranoid schizophrenia | schizophrenia |
| E1034 | Acute exacerbation of chronic paranoid schizophrenia | schizophrenia |
| E1035 | Paranoid schizophrenia in remission | schizophrenia |
| E103z | Paranoid schizophrenia NOS | schizophrenia |
| E104 | Acute schizophrenic episode | schizophrenia |
| E106 | Residual schizophrenia | schizophrenia |
| E106-1 | Restzustand - schizophrenia | schizophrenia |
| E107 | Schizo-affective schizophrenia | schizophrenia |
| E1070 | Unspecified schizo-affective schizophrenia | schizophrenia |
| E1071 | Subchronic schizo-affective schizophrenia | schizophrenia |
| E1072 | Chronic schizo-affective schizophrenia | schizophrenia |
| E1073 | Acute exacerbation subchronic schizo-affective schizophrenia | schizophrenia |
| E1074 | Acute exacerbation of chronic schizo-affective schizophrenia | schizophrenia |
| E1075 | Schizo-affective schizophrenia in remission | schizophrenia |
| E107z | Schizo-affective schizophrenia NOS | schizophrenia |
| E10y | Other schizophrenia | schizophrenia |
| E10y-1 | Cenesthopathic schizophrenia | schizophrenia |
| E10y0 | Atypical schizophrenia | schizophrenia |
| E10y1 | Coenesthopathic schizophrenia | schizophrenia |
| E10yz | Other schizophrenia NOS | schizophrenia |
| E10z | Schizophrenia NOS | schizophrenia |
| Eu20 | [X]Schizophrenia | bipolar |
| Eu200 | [X]Paranoid schizophrenia | schizophrenia |
| Eu200-1 | [X]Paraphrenic schizophrenia | schizophrenia |
| Eu201 | [X]Hebephrenic schizophrenia | schizophrenia |
| Eu201-1 | [X]Disorganised schizophrenia | schizophrenia |
| Eu202 | [X]Catatonic schizophrenia | schizophrenia |
| Eu202-1 | [X]Catatonic stupor | schizophrenia |
| Eu202-2 | [X]Schizophrenic catalepsy | schizophrenia |
| Eu202-3 | [X]Schizophrenic catatonia | schizophrenia |
| Eu202-4 | [X]Schizophrenic flexibilatis cerea | schizophrenia |
| Eu203 | [X]Undifferentiated schizophrenia | schizophrenia |
| Eu203-1 | [X]Atypical schizophrenia | schizophrenia |
| Eu204 | [X]Post-schizophrenic depression | schizophrenia |
| Eu205 | [X]Residual schizophrenia | schizophrenia |
| Eu205-1 | [X]Chronic undifferentiated schizophrenia | schizophrenia |
| Eu205-2 | [X]Restzustand schizophrenic | schizophrenia |
| Eu206 | [X]Simple schizophrenia | schizophrenia |
| Eu20y | [X]Other schizophrenia | schizophrenia |
| Eu20y-1 | [X]Cenesthopathic schizophrenia | schizophrenia |
| Eu20y-2 | [X]Schizophreniform disord NOS | schizophrenia |
| Eu20y-3 | [X]Schizophrenifrm psychos NOS | schizophrenia |
| Eu20z | [X]Schizophrenia, unspecified | schizophrenia |
| Eu232-4 | [X]Schizophrenic reaction | schizophrenia |
| ZV110 | [V]Personal history of schizophrenia | schizophrenia |
| 1464 | H/O: schizophrenia | schizophrenia |
| **Additional EMIS codes** | **Term** | **Group** |
| EMISQHY1 | Hypomanic | bipolar |
| EGTON118 | Obsessional compulsive psychosis | other psychosis |
| EMISCDE13 | Delusions | other psychosis |
| EMISICD10\|F2381 | Other acute and transient psychotic disorders, with associated acute stress | other psychosis |
| EMISQPA1 | Paranoid | other psychosis |
| EMISICD10\|F2018 | Hebephrenic schizophrenia, other | schizophrenia |
| EMISICD10\|F2035 | Undifferentiated schizophrenia, complete remission | schizophrenia |
| EMISICD10\|F2041 | Post-schizophrenic depression, episodic with progressive deficit | schizophrenia |
| EMISICD10\|F2054 | Residual schizophrenia, incomplete remission | schizophrenia |
| EMISICD10\|F2065 | Simple schizophrenia, complete remission | schizophrenia |
| EMISICD10\|F2098 | Schizophrenia, unspecified, other | schizophrenia |

# Supplementary Table 2: ICD-10 codes to determine primary diagnosis of first episode of an admission

| **Physical health admission** | **ICD-10** |
| --- | --- |
| Infections and parasitic disease | A00-B99 |
| Neoplasms | C00-D48 |
| Diseases of the blood | D50-D89 |
| Endocrine, nutritional and metabolic disease | E00-E90 |
| Diseases of the nervous system | G00-G99 |
| Diseases of the eye and adnexa | H00-H59 |
| Diseases of the ear and mastoid process | H60-H95 |
| Diseases of the circulatory system | I00-I99 |
| Diseases of the respiratory system | J00-J99 |
| Diseases of the digestive system | K00-K93 |
| Diseases of the skin and subcutaneous tissue | L00-L99 |
| Diseases of the musculoskeletal system and connective tissue | M00-M99 |
| Diseases of the genitourinary system | N00-N99 |
| Symptoms and signs involving the circulatory and respiratory system | R00-R09 |
| Symptoms and signs involving the digestive system and abdomen | R10-R19 |
| Symptoms and signs involving the skin and subcutaneous tissue | R20-R23 |
| Symptoms and signs involving the nervous and musculoskeletal systems | R25-R29 |
| Symptoms and signs involving the urinary system | R30-R39 |
| Fever of unknown origin NOS | R50 |
| Headache NOS | R51 |
| Pain NOS | R52 |
| Malaise and fatigue NOS | R53 |
| Syncope and collapse NOS | R55 |
| Convulsions NOS | R56 |
| Shock NOS | R57 |
| Enlarged lymph nodes NOS | R59 |
| Cachexia | R64 |
| Systemic inflammatory response syndrome | R65 |
| Hypothermia not associated with low environmental temperature | R68.0 |
| Elevated erythrocytes | R70 |
| Abnormality of red blood cells | R71 |
| Abnormality of white blood cells | R72 |
| Elevated blood glucose | R73 |
| Abnormal serum enzyme levels | R74 |
| Laboratory evidence of HIV | R75 |
| Other abnormal immunological findings in serum | R76 |
| Other abnormalities of plasma proteins | R77 |
| Other abnormalities of blood chemistry | R79 |
| Other abnormal findings in urine | R80.2 - R82.4; R82.6-R82.9 |
| Abnormal findings on examination of other bodily fluid | R83-R89 |
| Abnormal findings on diagnostic imaging/function studies | R90-R94 |
| New disease of uncertain aetiology | U00 -U 49 |
| Bacterial agent resistant to antibiotics | U80-U89 |
| General health check | Z00.0-Z00.3; Z00.6-Z00.8 |
| General health check | Z01 - Z02 |
| Observation for suspected TB | Z03.0 |
| Observation for suspected malignant neoplasm | Z03.1 |
| Other observation | Z03.3-Z03.5; Z03.8; Z03.9 |
| Malignant neoplasm follow up | Z08 |
| Follow up after radiotherapy | Z09.1 |
| Follow up after chemotherapy | Z09.2 |
| Health check of subpopulations | Z10 |
| Infectious disease screening | Z11 |
| Cancer screening | Z12 |
| Screening for other diseases | Z13.0-Z13.2; Z13.5-6; Z13.; Z13.9 |
| Infectious disease health hazard | Z20-Z29 |
| Prophylactic surgery | Z40 |
| Management of implanted device | Z45 |
| Dialysis | Z49 |
| Cardiac rehabilitation | Z50 |
| Radiotherapy | Z51.0 |
| Chemotherapy | Z51.1; Z51.2 |
| Desensitisation to allergens | Z51.6 |
| Convalescence following radiotherapy | Z54.1 |
| Convalescence following chemotherapy | Z54.2 |
| Dietary counselling | Z71.3 |
| HIV counselling | Z71.7 |
| Family history of cancer | Z80 |
| Personal history of cancer | Z85 |
| Personal history of other diseases | Z86.0-Z86.3; Z86.6-Z86.7 |
| Personal history of other diseases | Z87.0-Z87.4; Z87.6-Z87.8 |
| Personal history of allergy | Z88; Z91.0 |
| Transplant status | Z94 |
| Presence of cardiovascular implants and grafts | Z95 |
| **Accident, injuries and substance misuse** |  |
| Findings of drugs or other substances not normally found in blood | R78 |
| Injuries | S00-S99 |
| Injuries | T00-T14 |
| Foreign bodies | T15-T19 |
| Burns and corrosions | T20-T32 |
| Frostbite | T33-T35 |
| Poisoning | T36-T65 |
| Unspecified external cause | T66-T78 |
| Complications of trauma | T79 |
| Sequelae of injuries/poisoning | T90-98 |
| Accidents | V01-X59 |
| Intentional self-harm | X60-X84 |
| Assault | X85-Y09 |
| Undetermined intent | Y10-Y34 |
| War | Y35-Y36 |
| Sequelae of external causes | Y85-Y87 |
| Sequelae of external causes | Y89 |
| Evidence of alcohol involvement | Y90 -Y91 |
| Observation for suspected toxic effect of ingested substance | Z03.6 |
| Blood alcohol/drug observation | Z04.0 |
| Observation after injury | Z04.1-Z04.9 |
| Follow up after fracture | Z09.4 |
| Alcohol rehabilitation | Z50.2 |
| Drug rehabilitation | Z50.3 |
| Convalescence following treatment of fracture | Z54.4 |
| Alcohol counselling | Z71.4 |
| Drug counselling | Z71.5 |
| Tobacco counselling | Z71.6 |
| Problems related to lifestyle | Z72 |
| Personal history of substance misuse | Z86.4 |
| Personal history of self harm | Z91.5 |
| Personal history of other physical trauma | Z91.6 |
| **Mental health admission (excluded from analysis)** | Note - these include organic e.g. dementia. |
| Mental and behavioural disorders | F00-F99 |
| Psychiatric examination | Z00.4 |
| Observation for suspected mental and behavioural disorders | Z03.2 |
| Screening for mental and behavioural disorders | Z13.3 |
| Psychotherapy | Z50.4 |
| Convalescence following psychotherapy | Z54.3 |
| Personal history of mental disorder | Z86.5 |
| Personal history of psychological trauma | Z91.4 |
| **Potentially mental health related (exclude from analysis)** |  |
| Symptoms and signs involving cognition, perception, emotional states and behaviour | R40-R46 |
| Senility | R54 |
| Follow up after psychotherapy | Z09.3 |
| Screening for developmental disorders of childhood | Z13.4 |
| Problems related to life management difficulty | Z73 |
| **Maternity admissions and reproductive health (excluded from analysis)** |  |
| Pregnancy, childbirth, puerperium | O00-O99 |
| Certain conditions originating in the perinatal period | P00-P99 |
| Non specific symptoms peculiar to infancy | R68.1 |
| Circumstances related to reproduction | Z30-Z39 |
| Personal history of maternity complications | Z87.5-Z87.6 |
| **Congenital malformations, deformations and chromosomal anomalies (excluded from analysis)** |  |
|  | Q00-Q99 |
| Lack of normal physiological development | R62 |
| Screening for congenital | Z13.7 |
| Personal history of congenital | Z87.7 |
| **Other** |  |
| Speech and voice | R47-R49 |
| Haemorrhage NOS | R58 |
| Oedema NOS | R60 |
| Hyperhidrosis | R61 |
| Symptoms and signs concerning food and fluid intake | R63 |
| Dry mouth | R68.2 |
| Clubbing of nails | R68.3 |
| Other specified general symptoms and signs | R68.8 |
| Unknown and unspecified | R69 |
| Elevated urine levels of drugs, medicaments and biological substances | R82.6 |
| Ill defined and unknown cause of mortality | R95-R99 |
| Complications of surgery/medical care | T80-T88 |
| Complications of surgery/medical care | Y40-Y84 |
| Sequelae of surgical and medical care | Y88 |
| Nosocomial condition | Y95 |
| Work related condition | Y96 |
| Environmental pollution related condition | Y97 |
| Lifestyle related condition | Y98 |
| Examination of donor material | Z00.5 |
| Follow up after surgery | Z09.0 |
| Other follow up | Z09.7-Z09.9 |
| Surgery for non medical reason | Z41 |
| Correction to plastic surgery | Z42 |
| Attention to artificial openings | Z43 |
| Fitting prosthesis | Z44 |
| Fitting other device | Z46 |
| Other orthopaedic follow up | Z47 |
| Other surgical follow up care | Z48 |
| Other physical therapy | Z50.1 |
| Speech therapy | Z50.5 |
| Orthoptic therapy | Z50.6 |
| Occupational therapy | Z50.7 |
| Other rehabilitation | Z50.8; Z50.9 |
| Blood transfusion | Z51.3 |
| Preparatory care for other care | Z51.4 |
| Palliative care | Z51.5 |
| Other medical care | Z51.8; Z51.9 |
| Donor | Z52 |
| Procedure not carried out | Z53 |
| Convalescence following surgery | Z54.0 |
| Convalescence following other treatment | Z54.7-Z54.9 |
| Socioeconomic/psychosocial problems | Z55-Z65 |
| Sexual attitude/behaviour/orientation counselling | Z70 |
| Consulting on behalf of someone else | Z71.0 |
| Worried well | Z71.1 |
| Explanation of findings | Z71.2 |
| Other counselling | Z71.8-Z71.9 |
| Care dependent | Z74 |
| Awaiting appropriate facilities | Z75 |
| Other circumstances | Z76 |
| Family history of mental disorder | Z81 |
| Other family history of chronic disease | Z82-Z84 |
| Acquired absence of limb | Z89 |
| Acquired absence of organ | Z90 |
| Personal history of non compliance/poor hygiene/poor sleep/other | Z91.1-Z91.3; Z91.8 |
| Personal history of medical treatment | Z92 |
| Artificial opening status | Z93 |
| Presence of other implants | Z96-Z97 |
| Other post surgical states | Z98-99 |

# Supplementary Table 3: Model selection process

We compared unadjusted models using Poisson, then zero-inflated Poisson, negative binomial and zero inflated negative binomial for the two primary and two secondary outcomes. We found that zero-inflated models did not improve model fit substantially and given the easily interpretable nature of the negative binomial opted to use this throughout. Furthermore, zero-inflated models had issues converging when age was included as a continuous variable, and when year of index date was included. Even a reduced model did not converge for the demographic adjusted models of avoidable and accident, injury and substance misuse models.

| Admission type | Test | Poisson (P) | Zero-inflated Poisson (ZIP) | Negative binomial (NB) | Zero-inflated negative binomial (ZINB) |
| --- | --- | --- | --- | --- | --- |
| Unadjusted analysis with SMI vs no SMI as the exposure | | | | | |
| Planned physical | AIC | 304681.5 | 263160.5 | 192737.4 | 192741.5 |
|  | BIC | 304700.4 | 263198.2 | 192765.7 | 192788.6 |
|  | Vuong test | -- | ZIP superior to P p<0.0001 | NB superior to ZIP p<0.0001 | NB superior to ZINB  p<0.0001 |
| Emergency physical health | AIC | 213707 | 179206.1 | 152588.1 | 152577.6 |
|  | BIC | 213725.8 | 179243.8 | 152616.4 | 152624.8 |
|  | Vuong test | -- | ZIP superior to P  p<0.0001 | NB superior to ZIP  p<0.0001 | ZINB superior to NB:  Raw: p=0.04  NB similar to ZINB:  AIC-corrected: p=0.10 BIC-corrected: p=0.16 |
| Demographic-adjusted analysis, with SMI vs no SMI as the exposure, age as a categorical variable and with index year removed | | | | | |
| Planned physical | AIC | 281689.1 | 2498765 | 186327.7 | 186171.2 |
|  | BIC | 281896.5 | 250291.3 | 186544.6 | 186595.5 |
|  | Vuong test |  | ZIP superior to P  p<0.0001 | NB superior to ZIP p<0.0001 | ZINB superior to NB p<0.0001 |
| Emergency physical health | AIC | 190831.3 | 166454.2 | 144372.6 | 143925.2 |
|  | BIC | 191038.7 | 166869.1 | 144589.5 | 144349.5 |
|  | Vuong test |  | ZIP superior to P  p<0.0001 | NB superior to ZIP p<0.0001 | ZINB superior to NB:  p<0.0001 |

# Supplementary Table 4: Code lists to define face to face primary care consultations

| **CPRD Gold** |  |
| --- | --- |
| **Consultation code** | **Consultation Type** |
| 9 | Surgery consultation |
| 1 | Clinic |
| 33 | Triage |
| 39 | Medicine Management |
| 11 | Acute visit |
| 18 | Emergency Consultation |
| 3 | Follow-up/routine visit |
| 34 | Walk-in Centre |
| 36 | Co-op Surgery Consultation |
| 61 | Extended Hours |
| 40 | Community Clinic |
| **CPRD Aurum** |  |
| **Medcode ID** | **Consultation Type** |
| 1672871000006114 | GP Surgery |
| 1672851000006116 | Face to face consultation |
| 1809171000006114 | Routine consultation |
| 62211000000111 | Clinic note |
| 301141000000112 | Seen in GP unit |
| 1809161000006119 | Urgent consultation |
| 285368015 | Emergency appointment |
| 1809181000006112 | Emergency consultation |
| 1672621000006114 | Walk-in clinic |
| 1931821000006119 | Same day appointment |
| 1809191000006110 | Extended hours consultation |
| 1672881000006112 | Face to face consultation with relative/carer |
| 1850001000006116 | Follow up attendance face to face |
| 1928501000006119 | Seen in rapid access clinic at GP surgery |
| 241721000006114 | Patient initiated enc. NOS |
| 1839331000006117 | Seen in chronic obstructive pulmonary disease clinic |
| 1912191000006112 | Multidisciplinary team meeting with patient |
| 285223014 | Seen in diabetic clinic |

# Supplementary Figure 1: Flow chart of cohort inclusion/exclusion


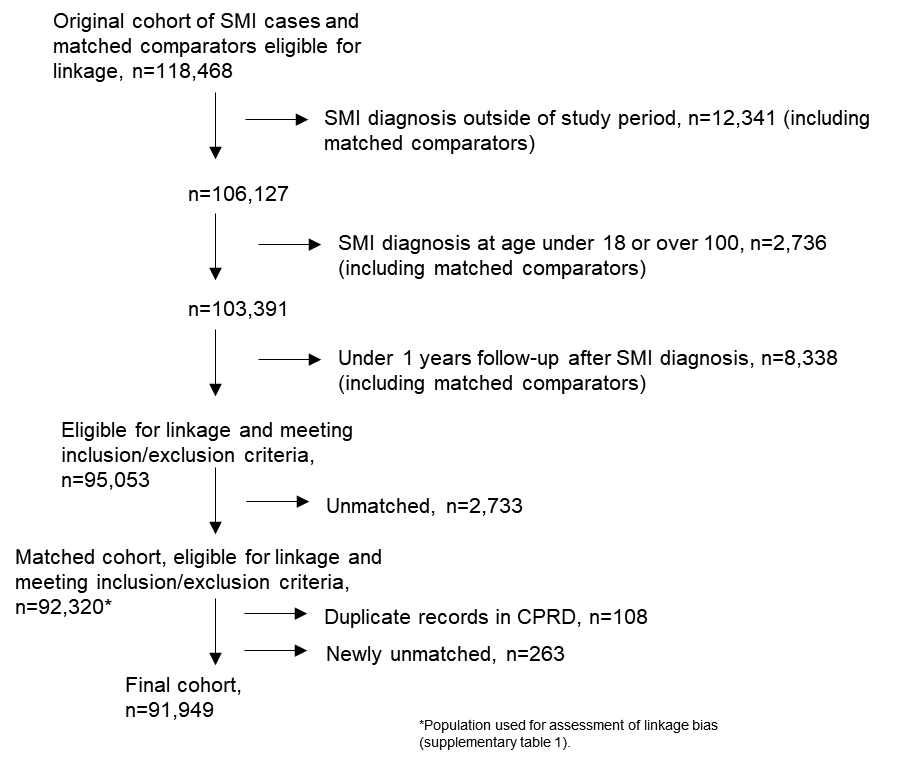


# Supplementary Table 5: Comparison of patients in CPRD eligible and ineligible for linkage with HES

|  | **Not eligible for linkage** | **Eligible for linkage** |
| --- | --- | --- |
| n (%) | 144,853 (61.07) | 92,320 (38.93) |
| **Data source** |  |  |
| CPRD Gold (%) | 13,052 (9.01) | 43,583 (47.21) |
| CPRD Aurum (%) | 131,801 (91.00) | 48,737 (52.79) |
| **SMI diagnosis (%)** |  |  |
| None | 113,269 (78.20) | 71,674 (77.64) |
| Schizophrenia | 7,314 (5.05) | 4,581 (4.96) |
| Bipolar | 11,385 (7.86) | 7,480 (8.10) |
| Other | 12,885 (8.90) | 8,585 (9.30) |
| **Age at diagnosis (median [IQR])** | 41.63 [30.13, 56.46] | 41.62 [29.72, 56.94] |
| **Female (%)** | 71478 (49.35) | 45,015 (48.76) |
| **Region (%)** | |  |
| East Midlands | 2,732 (1.89) | 3,084 (3.34) |
| East of England | 7,206 (4.97) | 8,015 (8.68) |
| London | 29,060 (20.06) | 18,086 (19.59) |
| North East | 8,415 (5.81) | 1,878 (2.03) |
| North West | 31,046 (21.43) | 12,219 (13.24) |
| South Central | 12,735 (8.79) | 12,663 (13.72) |
| South East Coast | 10,013 (6.91) | 9,008 (9.76) |
| South West | 13,899 (9.60) | 12,929 (14.00) |
| West Midlands | 23,832 (16.45) | 10,921 (11.83) |
| Yorkshire And The Humber | 5,915 (4.08) | 3,517 (3.81) |
| **Patient-level IMD (%)** | |  |
| 1 – least deprived | 2 (0.00) | 16,830 (18.23) |
| 2 | 3 (0.00) | 17,265 (18.70) |
| 3 | 2 (0.00) | 17,886 (19.37) |
| 4 | 3 (0.00) | 20,537 (22.25) |
| 5 – most deprived | 1 (0.00) | 19,655 (21.29) |
| Missing | 144,842 (99.99) | 147 (0.16) |
| **Ethnicity (%)** | |  |
| Asian | 7,258 (5.01) | 5,016 (5.43) |
| Black | 5,816 (4.02) | 3,157 (3.42) |
| Mixed | 1,552 (1.07) | 755 (0.82) |
| Other | 2,500 (1.73) | 2,259 (2.45) |
| White | 75,270 (51.96) | 42,163 (45.67) |
| Missing | 52,457 (36.21) | 38,970 (42.21) |
| **BMI category (heaviest ever) (%)** | |  |
| Underweight | 2,053 (1.42) | 1,278 (1.38) |
| Normal range | 37,753 (26.06) | 24,854 (26.92) |
| Overweight | 40,345 (27.85) | 26,216 (28.40) |
| Obese | 41,798 (28.86) | 25,862 (28.01) |
| Missing | 22,904 (15.81) | 14,110 (15.28) |
| **Smoking status (most recent ever) (%)** | |  |
| Current smoker | 47,009 (32.45) | 28,467 (30.84) |
| Ex-smoker | 46,671 (32.22) | 29,864 (32.35) |
| Never smoker | 42,659 (29.45) | 29,784 (32.26) |
| Missing | 8,514 (5.88) | 4,205 (4.55) |
| **Alcohol misuse (%)** | 8,514 (5.88) | 4,679 (5.07) |
| **Drug misuse (%)** | 6,246 (4.31) | 3,437 (3.72) |
| **Comorbidities (%)** |  |  |
| No comorbidities | 67,754 (46.77) | 43,223 (46.82) |
| One comorbidity | 35,276 (24.35) | 22,929 (24.84) |
| More than one comorbidity | 41,823 (28.87) | 26,168 (28.34) |
| **Deaths (%)** | 10,587 (7.31) | 6,648 (7.20) |
| **Age at death (median [IQR])** | 80.91 [67.96, 88.27] | 82.11 [69.76, 89.12] |
| **Follow up time (median [IQR])** | 4.90 [2.53, 9.05] | 4.74 [2.49, 8.50] |
| **Baseline time (median [IQR])** | 8.37 [2.51, 18.09] | 8.07 [2.35, 18.00] |

# Supplementary Table 6: Primary care consultations in the year prior to index and year after index

|  | **1 year prior to index** | | | | **1 year after index** | | | |
| --- | --- | --- | --- | --- | --- | --- | --- | --- |
|  | **No SMI** | **Schiz*** | **Bipolar** | **Other** | **No SMI** | **Schiz*** | **Bipolar** | **Other** |
| Do not have 1 year baseline | 7,829 (10.97) | 1,741 (38.24) | 1,842 (24.71) | 1,750 (20.44) |  |  |  |  |
| None | 20,835 (32.78) | 607 (21.59) | 804 (14.32) | 1,203 (17.67) | 23,474  (32.89) | 760 (16.69) | 836 (11.21) | 1,214 (14.18) |
| 1-5 | 25,587 (40.26) | 1,064 (37.84) | 1,396 (24.87) | 2,182 (32.04) | 28,661 (40.15) | 1,562 (34.31) | 1,789 (24.00) | 2,568 (30.00) |
| 6-10 | 9,816 (15.45) | 504 (17.92) | 1,304 (23.23) | 1,503 (22.07) | 10,856 (15.21) | 983 (21.59) | 1,781 (23.89) | 2,001 (23.38) |
| 11-20 | 5,668 (8.92) | 453 (16.11) | 1,441 (25.67) | 1,304 (19.15) | 6,450 (9.04) | 841 (18.47) | 2,030 (27.23) | 1,874 (21.89) |
| >20 | 1,646 (2.59) | 184 (6.54) | 668 (11.90) | 618 (9.07) | 1,940 (2.72) | 407 (8.94) | 1,019 (13.67) | 903 (10.55) |
| Median (IQR) | 2 (0-6) | 4 (1-9.25) | 8 (3-14) | 6 (2-12) | 2 (0-6) | 5 (2-11) | 8 (4-15) | 7 (2-13) |
| Max | 118 | 74 | 96 | 176 | 156 | 93 | 179 | 146 |

*Schiz: Schizophrenia

# Supplementary Table 7: Sensitivity analysis limiting follow up to the first year after diagnosis

|  |  | **Any SMI** | **Schizophrenia** | **Bipolar disorder** | **Other psychoses** |
| --- | --- | --- | --- | --- | --- |
| **Planned Physical health, IRR (95% CI)** | Crude | 1.06 (0.98-1.16) | 0.73 (0.60-0.89) | 1.21 (1.08-1.35) | 1.12 (0.99-1.26) |
|  | Demographic adjusted | 1.13 (1.04-1.23) | 0.94 (0.77-1.15) | 1.30 (1.17-1.45) | 1.07 (0.94-1.21) |
|  | Demographic, physical health and risk factor adjusted | 1.08 (0.99-1.18) | 0.93 (0.76-1.14) | 1.23 (1.10-1.37) | 1.03 (0.91-1.17) |
| **Emergency physical health, IRR (95% CI)** | Crude | 2.58 (2.39-2.77) | 2.05 (1.81-2.34) | 2.24 (2.01-2.51) | 3.14 (2.86-3.45) |
|  | Demographic adjusted | 2.63 (2.44-2.84) | 2.40 (2.10-2.74) | 2.49 (2.23-2.79) | 2.85 (2.58-3.15) |
|  | Demographic, physical health and risk factor adjusted | 2.38 (2.20-2.58) | 2.31 (2.02-2.66) | 2.26 (2.01-2.54) | 2.51 (2.27-2.77) |
| **Emergency accidents, injuries and substance misuse, IRR (95% CI)** | Crude | 6.25 (5.53-7.07) | 5.62 (4.09-7.72) | 5.97 (5.05-7.05) | 6.83 (6.06-7.70) |
|  | Demographic adjusted | 6.18 (5.46-6.98) | 5.81 (4.27-7.91) | 5.83 (4.96-6.85) | 6.65 (5.87-7.54) |
|  | Demographic, physical health and risk factor adjusted | 5.19 (4.57-5.89) | 4.94 (3.58-6.81) | 5.06 (4.31-5.95) | 5.41 (4.75-6.16) |
| **ACSC, IRR (95% CI)** | Crude | 2.86 (2.46-3.33) | 2.66 (2.05-3.46) | 2.26 (1.81-2.81) | 3.50 (2.80-4.36) |
|  | Demographic adjusted | 2.93 (2.51-3.43) | 3.15 (2.38-4.16) | 2.45 (1.97-3.05) | 3.23 (2.54-4.10) |
|  | Demographic, physical health and risk factor adjusted | 2.60 (2.23-3.03) | 3.11 (2.33-4.17) | 2.23 (1.76-2.82) | 2.68 (2.18-3.30) |
| **Non-ACSC emergency physical health, IRR (95% CI)** | Crude | 2.50 (2.31-2.71) | 1.90 (1.65-2.19) | 2.23 (1.98-2.51) | 3.06 (2.77-3.38) |
|  | Demographic adjusted | 2.54 (2.34-2.76) | 2.22 (1.92-2.57) | 2.48 (2.20-2.79) | 2.74 (2.47-3.04) |
|  | Demographic, physical health and risk factor adjusted | 2.32 (2.13-2.53) | 2.14 (1.84-2.49) | 2.25 (2.00-2.54) | 2.46 (2.21-2.73) |

# Supplementary Table 8: Sensitivity analysis investigating the impact of healthcare utilisation in the year prior to diagnosis, in those with one year baseline (n=79,080 (86.00%); SMI: 15,235 (74.07%); comparators: 63,845 (89.02%))

|  | **Adjusted for demographic, physical health and risk factors, plus:** | **Any SMI** | **Schizophrenia** | **Bipolar disorder** | **Other psychoses** |
| --- | --- | --- | --- | --- | --- |
| **Planned physical health, IRR (95% CI)** | None | 1.02 (0.96-1.08) | 0.79 (0.69-0.89) | 1.21 (1.12-1.30) | 0.95 (0.87-1.04) |
|  | Primary care | 0.84 (0.79-0.89) | 0.67 (0.58-0.76) | 0.97 (0.90-1.04) | 0.80 (0.74-0.87) |
|  | Secondary care | 0.99 (0.94-1.05) | 0.80 (0.70-0.90) | 1.19 (1.11-1.28) | 0.90 (0.83-0.98) |
|  | Primary and secondary care | 0.84 (0.79-0.89) | 0.69 (0.61-0.78) | 0.98 (0.91-1.05) | 0.78 (0.72-0.85) |
| **Emergency physical health, IRR (95% CI)** | None | 1.98 (1.87-2.09) | 1.76 (1.60-1.94) | 2.00 (1.83-2.18) | 2.05 (1.91-2.20) |
|  | Primary care | 1.69 (1.60-1.78) | 1.54 (1.41-1.69) | 1.63 (1.50-1.77) | 1.79 (1.67-1.93) |
|  | Secondary care | 1.74 (1.66-1.83) | 1.62 (1.48-1.78) | 1.77 (1.65-1.91) | 1.76 (1.65-1.88) |
|  | Primary and secondary care | 1.52 (1.44-1.59) | 1.45 (1.32-1.59) | 1.49 (1.38-1.61) | 1.56 (1.47-1.67) |
| **Emergency accidents, injuries and substance misuse, IRR (95% CI)** | None | 3.88 (3.61-4.17) | 3.53 (2.94-4.24) | 3.99 (3.59-4.43) | 3.92 (3.58-4.30) |
|  | Primary care | 3.44 (3.21-3.68) | 3.13 (2.64-3.71) | 3.45 (3.10-3.83) | 3.55 (3.25-3.89) |
|  | Secondary care | 3.39 (3.16-3.63) | 3.08 (2.53-3.76) | 3.55 (3.22-3.92) | 3.38 (3.11-3.67) |
|  | Primary and secondary care | 3.06 (2.87-3.27) | 2.80 (2.33-3.37) | 3.14 (2.84-3.46) | 3.11 (2.86-3.38) |
| **ACSC, IRR (95% CI)** | None | 2.42 (2.16-2.71) | 2.16 (1.81-2.58) | 2.54 (2.11-3.04) | 2.43 (2.11-2.79) |
|  | Primary care | 2.13 (1.90-2.38) | 1.96 (1.64-2.35) | 2.16 (1.79-2.60) | 2.17 (1.90-2.47) |
|  | Secondary care | 2.07 (1.87-2.28) | 1.93 (1.62-2.30) | 2.11 (1.83-2.44) | 2.08 (1.81-2.39) |
|  | Primary and secondary care | 1.85 (1.67-2.04) | 1.78 (1.48-2.13) | 1.85 (1.58-2.15) | 1.88 (1.65-2.14) |
| **Non-ACSC emergency physical health, IRR (95% CI)** | None | 1.27 (1.22-1.33) | 1.15 (1.07-1.24) | 1.22 (1.14-1.30) | 1.36 (1.28-1.45) |
|  | Primary care | 1.22 (1.16-1.27) | 1.11 (1.03-1.19) | 1.15 (1.08-1.22) | 1.31 (1.23-1.39) |
|  | Secondary care | 1.20 (1.15-1.25) | 1.11 (1.03-1.19) | 1.17 (1.10-1.24) | 1.27 (1.20-1.34) |
|  | Primary and secondary care | 1.16 (1.11-1.21) | 1.07 (1.00-1.15) | 1.11 (1.04-1.17) | 1.23 (1.16-1.30) |

# Supplementary Table 9: Sensitivity analysis excluding the first year of follow up after diagnosis and updating covariates to that point

|  |  | **Any SMI** | **Schizophrenia** | **Bipolar disorder** | **Other psychoses** |
| --- | --- | --- | --- | --- | --- |
| **Planned Physical health, IRR (95% CI)** | Crude | 0.94 (0.89-1.01) | 0.58 (0.52-0.65) | 1.16 (1.08-1.26) | 0.94 (0.86-1.03) |
|  | Demographic adjusted | 1.07 (1.00-1.13) | 0.78 (0.70-0.87) | 1.31 (1.22-1.41) | 0.99 (0.90-1.09) |
|  | Demographic, physical health and risk factor adjusted | 0.98 (0.93-1.04) | 0.74 (0.66-0.82) | 1.21 (1.12-1.30) | 0.90 (0.82-0.98) |
| **Emergency physical health, IRR (95% CI)** | Crude | 1.98 (1.89-2.08) | 1.60 (1.44-1.78) | 1.80 (1.68-1.94) | 2.38 (2.24-2.54) |
|  | Demographic adjusted | 2.27 (2.15-2.40) | 2.13 (1.87-2.44) | 2.31 (2.12-2.52) | 2.31 (2.15-2.49) |
|  | Demographic, physical health and risk factor adjusted | 1.93 (1.83-2.04) | 1.90 (1.67-2.16) | 1.95 (1.79-2.12) | 1.94 (1.81-2.07) |
| **Emergency accidents, injuries and substance misuse, IRR (95% CI)** | Crude | 3.94 (3.61-4.28) | 3.58 (2.72-4.70) | 3.66 (3.27-4.09) | 4.43 (4.00-4.91) |
|  | Demographic adjusted | 4.48 (4.12-4.88) | 4.58 (3.51-5.97) | 4.21 (3.79-4.68) | 4.68 (4.24-5.18) |
|  | Demographic, physical health and risk factor adjusted | 3.64 (3.33-3.97) | 3.81 (2.83-5.14) | 3.56 (3.20-3.96) | 3.61 (3.28-3.97) |
| **ACSC, IRR (95% CI)** | Crude | 2.38 (2.18-2.59) | 2.09 (1.75-2.48) | 2.30 (2.01-2.63) | 2.61 (2.33-2.94) |
|  | Demographic adjusted | 2.84 (2.56-3.16) | 2.64 (2.22-3.13) | 3.09 (2.58-3.70) | 2.73 (2.37-3.16) |
|  | Demographic, physical health and risk factor adjusted | 2.42 (2.18-2.70) | 2.40 (2.04-2.81) | 2.56 (2.13-3.07) | 2.31 (2.03-2.62) |
| **Non-ACSC emergency physical health, IRR (95% CI)** | Crude | 1.89 (1.80-1.98) | 1.48 (1.32-1.65) | 1.70 (1.58-1.83) | 2.31 (2.16-2.47) |
|  | Demographic adjusted | 2.13 (2.01-2.25) | 1.99 (1.70-2.32) | 2.13 (1.95-2.32) | 2.19 (2.03-2.37) |
|  | Demographic, physical health and risk factor adjusted | 1.83 (1.73-1.94) | 1.78 (1.54-2.06) | 1.82 (1.67-1.99) | 1.86 (1.73-2.00) |

# Supplementary Table 10: Sensitivity analysis adjusting individual physical health conditions

|  | **Adjusted for demographic and risk factors plus:** | **Any SMI** | **Schizophrenia** | **Bipolar disorder** | **Other psychoses** |
| --- | --- | --- | --- | --- | --- |
| **Planned Physical health, IRR (95% CI)** | Physical health (count) | 1.03 (0.98-1.09) | 0.79 (0.71-0.88) | 1.26 (1.17-1.35) | 0.95 (0.87-1.04) |
|  | Physical health (individual conditions) | 1.03 (0.97-1.08) | 0.79 (0.71-0.88) | 1.25 (1.17-1.34) | 0.94 (0.87-1.02) |
| **Emergency physical health, IRR (95% CI)** | Physical health (count) | 2.07 (1.97-2.18) | 2.03 (1.80-2.28) | 2.07 (1.91-2.23) | 2.09 (1.96-2.23) |
|  | Physical health (individual conditions) | 2.01 (1.91-2.12) | 1.98 (1.76-2.22) | 2.01 (1.87-2.17) | 2.03 (1.91-2.17) |
| **Emergency accidents, injuries and substance misuse, IRR (95% CI)** | Physical health (count) | 4.07 (3.75-4.41) | 4.12 (3.13-5.42) | 4.00 (3.62-4.42) | 4.10 (3.77-4.46) |
|  | Physical health (individual conditions) | 4.03 (3.72-4.37) | 4.08 (3.11-5.36) | 3.99 (3.61-4.40) | 4.05 (3.73-4.39) |
| **ACSC, IRR (95% CI)** | Physical health (count) | 2.55 (2.30-2.82) | 2.65 (2.25-3.12) | 2.57 (2.16-3.06) | 2.48 (2.17-2.83) |
|  | Physical health (individual conditions) | 2.39 (2.17-2.64) | 2.45 (2.10-2.86) | 2.44 (2.06-2.88) | 2.32 (2.05-2.62) |
| **Non-ACSC emergency physical health, IRR (95% CI)** | Physical health (count) | 1.92 (1.82-2.03) | 1.88 (1.65-2.14) | 1.96 (1.81-2.11) | 2.00 (1.87-2.14) |
|  | Physical health (individual conditions) | 1.92 (1.82-2.03) | 1.85 (1.62-2.11) | 1.92 (1.78-2.08) | 1.96 (1.83-2.10) |

# Supplementary Table 11: Sensitivity analysis to investigate the effect of using a zero-inflated negative binomial model

|  |  | **Any SMI** | **Schizophrenia** | **Bipolar disorder** | **Other psychoses** |
| --- | --- | --- | --- | --- | --- |
| **Zero inflated negative binomial model** | | | | | |
| **Planned Physical health, IRR (95% CI)** | Crude (OR of certain zero admissions) | 0.23 (0.19-0.29) | 0.0002 (0.0000-0.0005) | 16.36 (13.29-20.13) | 0.00002 (0.0001-0.0003) |
|  | Crude (IRR of admissions) | 0.97 (0.91-1.03) | 0.61 (0.54-0.68) | 1.18 (1.10-1.28) | 0.97 (0.89-1.06) |
|  | Demographic adjusted (OR of certain zero admissions) | 0.44 (0.23-0.84) | 0.09 (0.004-2.19) | 0.50 (0.23-1.09) | 0.33 (0.10-1.08) |
|  | Demographic adjusted (IRR of admissions) | 1.04 (0.97-1.11) | 0.73 (0.64-0.83) | 1.24 (1.15-1.34) | 0.98 (0.89-1.08) |
| **Emergency physical health, IRR (95% CI)** | Crude (OR of certain zero admissions) | 0.0048 (0.0016-0.0141) | 0.0000 (0.0000-0.0000) | 0.0002 (0.0000-0.0004) | 0.055 (0.0000-1.86e+11) |
|  | Crude (IRR of admissions) | 2.01 (1.90-2.12) | 1.61 (1.44-1.79) | 1.81 (1.67-1.97) | 2.39 (2.20-2.60) |
|  | Demographic adjusted (OR of certain zero admissions) | 0.67 (0.52-0.86) | 0.60 (0.39-0.95) | 0.49 (0.31-0.80) | 0.83 (0.59-1.17) |
|  | Demographic adjusted (IRR of admissions) | 2.14 (2.02-2.28) | 1.93 (1.69-2.20) | 2.11 (1.91-2.33) | 2.27 (2.09-2.47) |
| **Negative binomial model** | | | | | |
| **Planned Physical health, IRR (95% CI)** | Crude (IRR of admissions) | 0.97 (0.91-1.03) | 0.61 (0.54-0.68) | 1.18 (1.10-1.28) | 0.97 (0.89-1.06) |
|  | Demographic adjusted (IRR of admissions) | 1.07 (1.00-1.13) | 0.78 (0.70-0.87) | 1.28 (1.19-1.37) | 1.02 (0.93-1.12) |
| **Emergency physical health, IRR (95% CI)** | Crude (OR of certain zero admissions) | 2.08 (2.00-2.17) | 1.68 (1.52-1.85) | 1.89 (1.76-2.03) | 2.49 (2.34-2.64) |
|  | Demographic adjusted (IRR of admissions) | 2.31 (2.20-2.43) | 2.09 (1.86-2.33) | 2.34 (2.17-2.54) | 2.39 (2.23-2.57) |

*Note, age is categorised as three age groups, and calendar year of index date is not controlled for due to lack of model convergency for the zero-inflated negative binomial model
